# The fast milk acidifying phenotype of *Streptococcus thermophilus* can be acquired by natural transformation of the genomic island encoding the cell-envelope proteinase PrtS

**DOI:** 10.1186/1475-2859-10-S1-S21

**Published:** 2011-08-30

**Authors:** Damien Dandoy, Christophe Fremaux, Marie Henry de Frahan, Philippe Horvath, Patrick Boyaval, Pascal Hols, Laetitia Fontaine

**Affiliations:** 1 Biochimie et Génétique Moléculaire Bactérienne, Institut des Sciences de la Vie, Université catholique de Louvain, Place Croix du Sud 5/L7.07.06, B-1348 Louvain-la-Neuve, Belgium; 2 Danisco France SAS, BP 10, 86220 Dangé-Saint-Romain, France

## Abstract

**Background:**

In industrial fermentation processes, the rate of milk acidification by *Streptococcus thermophilus* is of major technological importance. The cell-envelope proteinase PrtS was previously shown to be a key determinant of the milk acidification activity in this species. The PrtS enzyme is tightly anchored to the cell wall via a mechanism involving the typical sortase A (SrtA) and initiates the breakdown of milk casein into small oligopeptides. The presence or absence of PrtS divides the *S. thermophilus* strains into two phenotypic groups i.e. the slow and the fast acidifying strains. The aim of this study was to improve the milk acidification rate of slow *S. thermophilus* strains, and hence optimise the fermentation process of dairy products.

**Results:**

In the present work, we developed for the first time a strategy based on natural transformation to confer the rapid acidification phenotype to slow acidifying starter strains of *S. thermophilus*. First, we established by gene disruption that (i) *prtS*, encoding the cell-envelope proteinase, is a key factor responsible for rapid milk acidification in fast acidifying strains, and that (ii) *srtA*, encoding sortase A, is not absolutely required to express the PrtS activity. Second, a 15-kb PCR product encompassing the *prtS* genomic island was transfered by natural transformation using the competence-inducing peptide in three distinct *prtS*-defective genetic backgrounds having or not a truncated sortase A gene. We showed that in all cases the milk acidification rate of transformants was significantly increased, reaching a level similar to that of wild-type fast acidifying strains. Furthermore, it appeared that the *prtS*-encoded activity does not depend on the *prtS* copy number or on its chromosomal integration locus.

**Conclusion:**

We have successfully used natural competence to transfer the *prtS* locus encoding the cell-envelope proteinase in three slow acidifying strains of *S. thermophilus*, allowing their conversion into fast acidifying derivatives. The efficient protocol developed in this article will provide the dairy industry with novel and optimised *S. thermophilus* starter strains.

## Introduction

Lactic acid bacteria (LAB) are widely used as starter cultures in the manufacture of dairy products due to their efficient utilisation of milk constituents, principally lactose and caseins. Their capacity to produce lactic acid as the main metabolic end-product of lactose fermentation is of major economic importance, since acidification inhibits the growth of spoilage organisms. Previous studies highlighted a link between the presence of an efficient casein proteolytic system and fast growth and acidification rate of LAB in milk [1]. Casein breakdown is initiated by the cell-envelope proteinases (CEPs) and the resulting oligopeptides are then transported into the cell where they are further hydrolysed by a set of various intracellular peptidases [2,3]. LAB possess generally only one CEP, but some strains of *Lactobacillus delbrueckii* subsp. *bulgaricus* present two or more CEP-encoding genes [4-6]. Similar to many surface proteins such as adhesins, CEPs generally contain a C-terminal LPXTG motif and are anchored to the cell wall via a mechanism involving the typical sortase A (SrtA) in Gram-positive bacteria [3,7]. Transpeptidation of LPXTG proteins by the membrane-bound SrtA involves two steps: (i) a cleavage reaction inside the LPXTG motif, and (ii) covalent attachment of the processed and exported N-terminal form to the pentapeptide chain of one peptidoglycan repetition unit [8,9].

**Figure 1:**
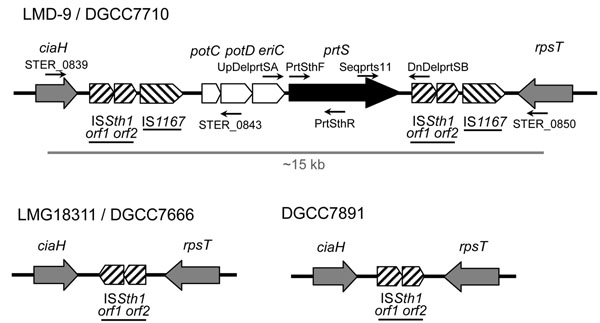
**Schematic representation of the *ciaH-rpsT* locus of *S. thermophilus.*** LMD-9 and DGCC7710 are PrtS^+^ strains. LMG18311, DGCC7666 and DGCC7891 are PrtS^-^ strains. Genes encoding proteins are represented by thick arrows; insertion sequences (striped), genes conserved between strains (grey). The following elements are indicated: name of the insertion sequences (underlined), region amplified by PCR to transfer the *prtS* locus from DGCC7710 to LMG18311, DGCC7891 and DGCC7666 (thick grey line), primers used to amplify the *prtS* regions and to validate the identity of PrtS^+^ and PrtS^-^ strains (thin black arrows).

S*treptococcus thermophilus* is a thermophilic LAB which is considered as the second most important industrial dairy starter culture after *Lactococcus lactis*[*10*]. The CEP from *S. thermophilus*, PrtS, is a LPXTG-containing serine proteinase of the subtilisin family, similar to the cell envelope protease from other LAB [7,11,12]. In monoculture, PrtS is essential for rapid growth of *S. thermophilus* in milk and therefore confers a competitive advantage compared to PrtS-deficient strains [1]. However despite its relevant role for growth in milk, only 21 strains among the 135 strains of the INRA historical collection displayed a high level of proteinase activity, indicating that this characteristic is not common in this species [13]. Since *S. thermophilus* is generally found in mixed cultures, growth of PrtS-deficient strains relies on the use of oligopeptides released by other LAB such as *L. delbrueckii* subsp. *bulgaricus*, *Lactobacillus helveticus* or *L. lactis*[1]. However, during the last 10 years, this important metabolic trait for milk adaptation was drastically selected by the dairy industry, resulting in a strong increase of PrtS^+^ strains in industrial dairy products [14,15]. These probably result from the attractive technological implications associated with PrtS^+^ strains, such as optimal development in milk containing high protein content, rapid milk acidification, and acceleration of cheese ripening. Recently, Delorme and co-workers showed that in *S. thermophilus*, *prtS* is located in a 15-kb genomic island that was probably acquired by horizontal gene transfer [13]. Indeed, this region which consists of three open reading frames (ORF) present upstream of *prtS* is flanked by tandem repeats of IS elements (Fig. 1).

Besides the fast acidifying phenotype, some *S. thermophilus* strains harbour other industrially relevant phenotypes such as texturing properties, bacteriocins production, and phage resistance. However, these features are rarely found together in a single starter strain. Our objective was to develop a strategy to transfer the rapid acidification phenotype in different genetic backgrounds. In a first step, we defined the minimum genetic information associated with this phenotype by investigating the contribution of PrtS and Sortase A. In a second step, we took advantage of competence to transfer *prtS* from the fast-acidifying strain DGCC7710 into three different slow strains. The PrtS-positive transformants were characterised at the genetic level by PCR mapping and at the phenotypic level by performing proteinase plate assays and monitoring milk acidification.

## Materials and methods

### Bacterial strains and growth conditions

The *S. thermophilus* strains used in this study are listed in Table 1. Strains were grown at 37°C or at 40°C. For natural transformation experiments, M17 broth (Difco Laboratories Inc., Detroit, MI) and reconstituted CDM [16] were used. These media were supplemented with 1% (wt/vol) of lactose (M17L or CDML, respectively). Two different skimmed milks were used to assess the proteinase phenotype of strains: 10% (vol/vol) UHT milk (Candia GrandLait, Candia, France) and reconstituted 9% (wt/vol) milk (Humana Milchunion, Germany). Solid agar plates were typically prepared by adding 2% (w/v) agar to the medium. When necessary, 5 µg ml^-1^ chloramphenicol was added to the media. Inoculated solid plates, solely, were incubated in anaerobic conditions (BBL GasPak Systems, Becton Dickinson, Franklin lakes, NJ).

**Table 1:** Characteristics of *S. thermophilus* strains used in this work

Strain	Phenotype		Genotype		Transformation rate
		
	F	T	*prtS*	*strA*	**(+ 1mM ComS17-24)**[20]
LMG18311	F^-^	T^+^	-	Trunc.	1.3 x 10^-1^
CNRZ1066	F^-^	T^+^	-	Trunc.	
LMD-9	F^+^	T^-^	+	+	5.4 x 10^-3^
DGCC7790	F^-^	T^+^	-	+	
DGCC7710	F^+^	T^+^	+	+	1.0 x 10^-2^
DGCC7853	F^+^	T^-^	+	+	
DGCC7879	F^+^	T^-^	+	+	
DGCC715	F^+^	T^-^	+	+	
DGCC7773	F^-^	T^+^	-	Trunc.	
DGCC7785	F^-^	T^+^	-	Trunc.	
DGCC7796	F^+^	T^-^	+	+	
DGCC7854	F^+^	T^-^	+	+	
DGCC7891/ND03	F^-^	T^+^	-	+	1.4 x 10^-4^
DGCC7666	F^-^	T^-^	-	Trunc.	4.2 x 10^-2^
DGCC7694	F^-^	T^-^	-	+	
DGCC7809	F^+^	T^-^	+	+	
DGCC7909	F^-^	T^+^	-	Trunc.	
DGCC7984	F^+^	T^+^	+	+	
DGCC47	F^-^	T^+^	-	Trunc.	
DGCC7766	F^-^	T^-^	-	+	
DGCC855	F^+^	NT	+	+	
DGCC2057	F^+^	T^+^	+	+	
DGCC2058	F^-^	T^+^	-	+	
DGCC9791	F^-^	NT	-	+	
DGCC7856	F^-^	T^-^	-	+	
DGCC8014	F^+^	T^+^	+	+	

All strains are from Danisco collection, excepted strains LMD-9 (ATCC collection), CNRZ1066 (CNRZ collection), ND03[29] and LMG18311 (LMG collection)

For phenotype, F^+^ and F^-^ respectively refer to fast and slow acidifying strains when grown in pasteurized milk, T^+^ and T^-^ respectively refer to the presence and absence of texturing properties when grown in pasteurized milk, and NT for not tested.

For genotype, + indicates that the gene is detected and entire, – indicates that *prtS* is no detected, Trunc. indicates a truncated version of *srtA*

### Proteinase plate assay

The PrtS proteinase phenotype of *S. thermophilus* strains was determined on bacterial colonies grown on Fast Slow Difference Agar (FSDA) medium after 48 to 72 h of incubation at 37°C, as previously reported [17]. This medium contains 1.5% (wt/v) agar, 1.9% (wt/v) Sodium glycerophosphate (Prolabo, Merck, West Chester, PA), 10% (vol/vol) UHT half-skimmed milk (Candia GrandLait, Candia, France) and 0.001% (wt/v) of the bromocresol purple (BCP) indicator (Prolabo, Merck, West Chester, PA). On FSDA plates, bacteria possessing a proteinase activity appear as yellow, big, opaque colonies surrounded by a yellow area (fast acidifying phenotype), whereas proteinase-negative colonies appear small, flat, and translucent (slow acidifying phenotype).

### Determination of the kinetics parameters of acidification

The acidification activity of *S. thermophilus* strains was evaluated by calculating the maximum acidification rate and time necessary to reach pH 5.2. Practically, reconstituted 10% (wt/vol) UHT skimmed milk (autoclaved during 10 minutes, 110°C) was inoculated with 2% (vol/vol) of a 5ml-culture of *S. thermophilus* grown at 37°C during 24h in M17L broth. After 24h of incubation at 37°C, the cultures were inoculated at 1% (vol/vol) in 150ml skimmed milk i.e. either 10% (vol/vol) UHT milk (Candia GrandLait) or 9% reconstituted (wt/vol) milk (Humana Milchunion, Germany). The cultures were then incubated in a water bath at 40°C and the pH (pH electrode Mettler 405 DPAS SC, Toledo, Spain) was monitored during 16 hours using the CINAC system (Alliance Instruments, France) as previously described [18,19]. The pH was measured every second and values obtained during 5 minutes were averaged. The maximum acidification rate (V_m_), defined as the maximum slope of the pH curve (dpH/dt), was calculated using the CINAC v2.07 software and expressed as pH units/minute.

### Natural transformation experiments

Transformation experiments were performed as previously described [20,21]. Briefly, an overnight culture of *S. thermophilus* grown in M17L at 37°C was washed twice (5,000 × *g*, 9 min, room temperature) in one volume of CDML and resuspended in one volume of CDML*.* The washed culture was then 30-fold diluted in CDML and DNA was added to small volumes (300 μl). The amount of DNA used was either 25 ng (purified overlapping PCR products) or 10 μg (PCR product encompassing the *prtS* genomic island). After 1h30 of incubation at 37°C, 1 μM of peptide ComS_17-24_ (purity >95%; supplied by Peptide 2.0 (Chantilly, VA)) was added to the culture. After 5 hours, samples (100 μl of serial dilutions in M17 broth) containing DNA, or not (negative control), were spread on selective plates and incubated anaerobically at 37°C. These plates contained M17L broth supplemented with 5 μg/ml chloramphenicol (transformation of *lox66-*P32-*cat-lox71*-containing PCR products) or FSDA medium (transformation of the *prtS* genomic island). In the latter case, 10^8^ cells were plated on FSDA dishes. After 72 hours of incubation, colonies displaying a fast acidifying phenotype (see above) i.e. colonies emerging on the lawn of slow acidifying colonies were recovered and isolated on FSDA plates. The presence of *prtS* in fast acidifying colonies was then validated by PCR extension.

### DNA techniques

For general molecular biology techniques, we followed the instructions given by Sambrook et al. [22]. Preparation of *S. thermophilus* chromosomal DNA was performed as described previously [23]. The primers used in this study were purchased from Eurogentec (Seraing, Belgium) and are listed in Table S1 of the Additional file 1. PCRs were performed with Fhusion high-fidelity DNA polymerase (Finnzymes Espoo, Finland) or LA Taq™ Polymerase (Takara bio, Otsu, Japan) in a GeneAmp PCR system 2400 (Applied Biosystems, Foster City, CA).

### Construction of *prtS* and *srtA* deletion mutants

Mutant strains (*∆srtA*::*lox66*-P32-*cat*-*lox72* and *∆prtS*::*lox66*-P32-*cat*-*lox72*) of LMD-9 and DGCC7710 were constructed by replacing the sequence between the start and stop codons of *srtA* and *prtS* with the chloramphenicol resistance cassette *lox66-*P32-*cat-lox71* according to the procedure described by Fontaine et al. [20]. The primers used to construct these strains are listed in Table S1 of the Additional file 1.

## Results and discussion

### Fast acidifying strains contain the *prtS* genomic island and a full-length *srtA* gene

The strategy chosen to optimise the acidification rate of *S. thermophilus* strains consists of transferring the minimal genetic requirements associated with the fast acidifying phenotype. Based on previous knowledge on the functional role of PrtS and Sortase A in the acidification rate [1,11,12] and activity of LPXTG-surface proteins [24-26], respectively, we focused our attention on the co-occurrence of *srtA* and *prtS* in fast strains. For this purpose, we analysed a sample of fast and slow acidifying strains of *S. thermophilus*. Their phenotype was determined by a FSDA plate assay (Data not shown and [20]). They were selected by industrial manufacturers for their relevant phenotypes related to milk fermentation [20].

The presence of *prtS* was firstly analysed by PCR using primers specific to the *prtS* ORF or flanking genes (Additional file 1, Table S1). These primers were designed based on the sequence of the *prtS* genomic island in strain LMD-9. Amplification products of the expected size were obtained for 11 out of the 26 strains tested (22 strains plus the 4 sequenced strains LMD-9, LMG18311, CNRZ1066 and ND03) (Data not shown). As expected, the presence or absence of *prtS* was in perfect agreement with the fast or slow acidifying phenotypes of strains on FSDA medium, respectively (Table 1). In addition, the systematic finding of a PCR amplification product with primers targeting the two genes flanking *prtS* strongly suggests that its genetic context is similar to that described by Delorme and co-workers (Additional file 1, Fig. S1) [13]. Next, the 11 *prtS* ORFs were sequenced and the corresponding proteins were aligned with known sequences from strains LMD-9, CNRZ385 and JIM8232 (Additional file 1, Fig. S2). PrtS sequences share a high level of conservation at the protein level (between 96% and 100% of identity in pairwise alignments), which suggests a recent acquisition as hypothesised by Delorme et al. [13]. However, the phylogenetic tree deduced from the multiple alignment of PrtS sequences shows that they form three distinct groups (Additional file 1, Fig. S1). Each cluster is similarly represented, which suggests that at least three different sub-populations have emerged since the initial acquisition of the *prtS* locus.

A conserved LPNTG sorting motif was identified in the C-terminus part of all PrtS proteins (Additional file 1, Fig. S2), supporting our hypothesis of a putative role for Sortase A in PrtS activity of fast isolates. Consequently, the *srtA* ORFs were PCR-amplified from the 22 strains, sequenced and compared to *srtA* from strains LMD-9 (*ster_1255*), LMG18311 (*stu1277-stu1278*), CNRZ1066 (*str1277-str1278*) and ND03 (*STND1228*). The SrtA proteins are more divergent than PrtS proteins since they share between 93% and 100% identity (deduced from pairwise alignments) (Additional file 1, Figs. S3 and S4). We found that *srtA* from 7 out of 26 strains contains the same nonsense mutation that shortens the ORF size (378 bp instead of 758 bp) (Additional file 1, Fig. S4). The Y123STOP mutation probably abolishes SrtA activity in those strains. Interestingly, all PrtS^+^ isolates encode a full-length *srtA* ORF. Their SrtA protein also contains the conserved catalytic residues (H147, C212 and R220), essential for sortase activity [8,9]. The co-occurrence of a full-length *srtA* and *prtS* in fast acidifying isolates strongly suggests that these two components are both required for an optimal fast acidifying phenotype in *S. thermophilus*.

### PrtS is essential for fast milk acidification while SrtA is not required

**Figure 2:**
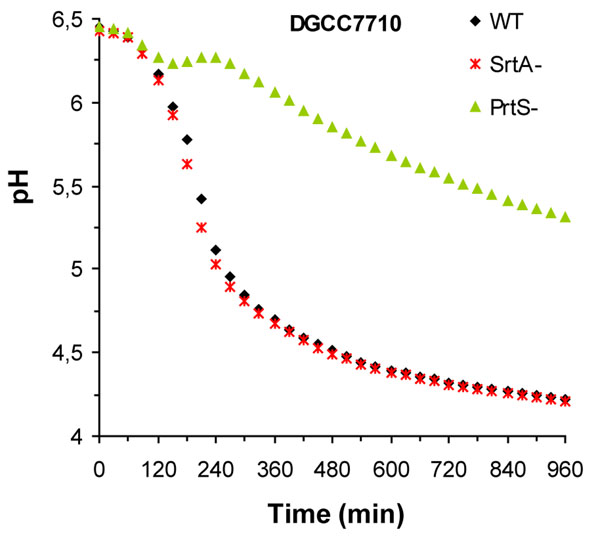
**Milk acidification kinetics of *srtA* and *prtS* mutants.** Milk acidification curves of the parental strain DGCC7710 (black diamonds), and its respective SrtA^-^ (*∆srtA*::*lox66*-P32-*cat*-*lox71;* red crosses) and PrtS^-^ (*∆prtS*:: *lox66*-P32-*cat*-*lox71;* green triangles) derivatives. All experiments were performed in the reconstitued milk at 40°C.

The individual contribution of SrtA and PrtS proteinase on milk acidification was investigated by replacing the corresponding ORFs in fast strain LMD-9 and DGCC7710 by a chloramphenicol resistance marker. The physiological effects in milk-based medium were investigated by plating the parental and mutant strains on FSDA medium and by measuring their acidification rates in UHT skimmed milk. As expected, both *∆prtS*::*lox66*-P32-*cat*-*lox71* derivative strains displayed the typical phenotype of slow acidifying strains on FSDA medium. Surprisingly, no striking morphological difference was observed between colonies of WT and *∆srtA*::*lox66*-P32-*cat*-*lox71* derivatives (Data not shown). These results were confirmed by monitoring the pH and measuring the acidification kinetics of strains growing in two skimmed milks: UHT and reconstituted milk. Similar acidification rates were obtained for wild-type and SrtA-defective strains, while *prtS*-negative mutants were severely impaired in their acidification capacities (Data shown for DGCC7710 and its derivatives in Fig. 2). Altogether, our results show that *prtS* is the predominant genetic determinant required for the fast acidification phenotype of *S. thermophilus* in milk-based culturing conditions.

### Isolation of fast acidifying PrtS^+^ transformants deriving from three slow strains

The transfer of *prtS* from fast to slow strains was achieved using the natural competence protocol that was recently developed [20]. The model strains selected for the proof of concept were DGCC7710 as donor, and LMG18311, DGCC7666 and DGCC7891 as receivers. These latter strains were chosen for different reasons: (i) they encode, or not, a full-length *srtA* ORF (Table 1), (ii) they display different transformation rates [20], (iii) they display different industrially-relevant properties [20], and (iv) their genome has been sequenced (draft genome for DGCC7666, unpublished data; and DGCC7891 is closely related to the recently sequenced strain ND03) [29]. A 15,283-kb PCR product encompassing the whole *prtS* island (between *ster_0839* and *ster_0850*) (Fig.1) from strain DGCC7710 was used as donor DNA. The entire region was sequenced and shares 99 % identity with the *prtS* region of LMD-9. The IS elements (IS*Sth1* and IS*1167*) flanking the *prtS* locus would mediate double homologous recombination events between the PCR-amplified DNA fragment and the chromosome of receiver strains. Indeed, these elements are widespread among *S. thermophilus* genomes (Table 2; Additional file 1, Fig. S3). The natural competence experiments were performed in the presence of 10 µg of the purified PCR fragment and transformants displaying a fast acidifying phenotype were recovered on FSDA plates. After three successive rounds of isolation on FSDA plates, the stable acquisitions of the *prtS* genomic island in fast acidifying clones were confirmed by PCR mapping. The integration site(s) of the *prtS* island were then determined by PCR on 3 to 5 clones per strain using a primer specific to IS*Sth1* or IS*1167* flanking genes, and the *prtS* locus (Additional file 1, Table S1). A random insertion of the *prtS* locus in both IS*Sth1* and IS*1167* loci was detected among strains and isolated clones (Table 2). Multiple insertions were also observed, reaching in some cases up to seven copies of *prtS* in the same clone (Table 2). The stable maintenance of *prtS* copies in three clones per strain was then studied in liquid milk-based medium. After ~160 generations, the copy number and the insertion profile determined by PCR mapping remained unchanged in all isolates (Data not show).

**Table 2:** Distribution of *prtS* loci among LMG18311, DGCC7666 and DGCC7891 genomes

Strains	Putative insertion sites and their surrounding genes													*prtS* insertion number
		
	IS*Sth1 orf1/2*						IS*1167*							
			
	*ciaH*	*blpT*	*galU*	*msrA1*	*topA*	*STND0510*	*stu0861*	*stu1089*	*STND0227*	*STND0823*	*STND0900*	*STND1130*	*STND1212*	
	*rpsT*	*stu1680*	*stu1836*	*brnQ*	*stu0900*	*STND0513*	*stu0868*	*stu1075*	*STND0229*	*STND-0825*	*STND0902*	*STND1132*	*STND1214*	
DGCC7666 PrtS^+^														
CL1	-	+	-	+	/	/	-	+	/	/	/	/	/	3
CL2	+	+	-	-	/	/	+	+	/	/	/	/	/	4
CL3	+	-	-	+	/	/	-	-	/	/	/	/	/	2
CL4	-	-	+	-	/	/	-	-	/	/	/	/	/	1
CL6	-	-	-	+	/	/	-	-	/	/	/	/	/	1
LMG18311 PrtS^+^														
CL3A	-	+	-	+	-	/	-	-	/	/	/	/	/	2
CL6A, 10A	+	-	-	-	-	/	-	-	/	/	/	/	/	1
CL9A, 17A	-	-	-	+	-	/	-	-	/	/	/	/	/	1
CL21A, 23A	-	-	+	-	-	/	-	-	/	/	/	/	/	1
DGCC7891 PrtS^+^														
CL1A, 19A	+	+	+	/	/	-	/	/	+	+	+	-	+	7
CL1B	+	+	+	/	/	-	/	/	+	+	-	-	+	6

Symbols: / , IS element not detected in the chromosome; - , *prtS* locus not detected; + , *prtS* locus detected

### Stable acquisition of the *prtS* genomic island improves the acidification activity of *S. thermophilus*

**Figure 3:**
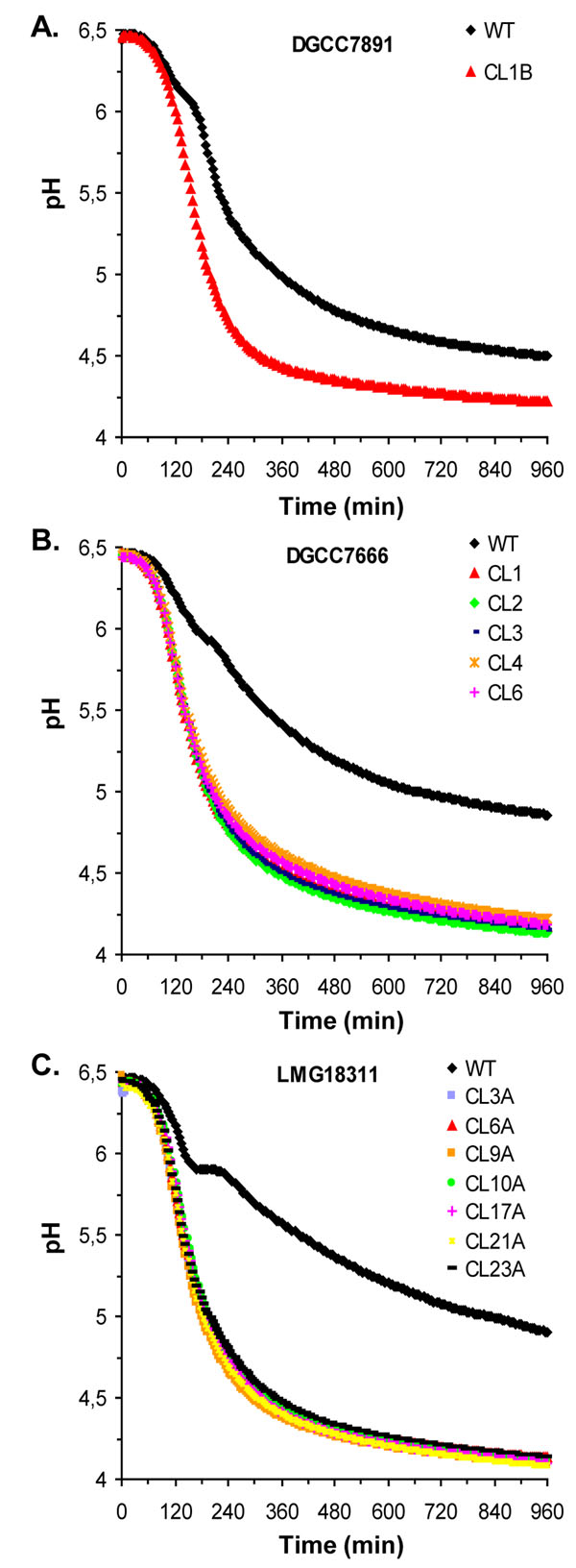
**Milk acidification kinetics of PrtS^+^ transformants.** Milk acidification curves of parental strains and their respective PrtS^+^ derivatives for DGCC7891 (A), DGCC7666 (B), and LMG18311 (C). All experiments were realised in the reconstituted milk at 40°C.

To further characterise the phenotype of PrtS^+^ derivative strains, their performance was analysed by determining kinetics parameters of acidification in milk. We tested culture conditions that are mimicking those encountered in industrial fermentation processes i.e. in the 10% UHT skimmed milk (vol/vol), and the 9% (wt/vol) reconstituted skimmed milk. The results are presented in Fig. 3 and in Table 3. Compared to the parental strains, acidification rates of all derivatives were remarkably improved (2.4 fold) in both milks. In addition, we observed a reduction of the latency period i.e. the time before the pH begins to decrease (Fig. 3 and Data not shown). Consequently, a significantly shorter fermentation time is needed to obtain pH 5.2 (2 to 3-fold) (Table 3). Interestingly, the acidification curves obtained in both milks were almost identical among all PrtS^+^ clones deriving from the same parental strain. They were also similar to natural fast acidifying strains DGCC7710 or LMD-9 (Table 3 and Data not shown).

**Table 3:** Acidifying capacities of *S. thermophilus* in milk

Strains	UHT milk		Reconstituted milk	
		
	Vmax	pH 5.2	Vmax	pH 5.2
	(pH U x 10^-4^ /min)	(min)	(pH Ux 10^-4^ /min)	(min)
DGCC7666	48	656	52	475
PrtS^+^ CL1	125	291	143	169
PrtS^+^ CL2	114	295	142	172
PrtS^+^ CL3	109	276	141	169
PrtS^+^ CL4	116	290	150	181
PrtS^+^ CL6	132	266	143	174
LMG18311	51	713	81	605
PrtS^+^ CL3A	122	284	159	164
PrtS^+^ CL6A	106	276	148	164
PrtS^+^ CL9A	125	277	156	159
PrtS^+^ CL10A	111	285	143	173
PrtS^+^ CL17A	102	379	155	167
PrtS^+^ CL21A	126	261	157	161
PrtS^+^ CL23A	128	296	158	168
DGCC7666	71	460	107	281
PrtS^+^ CLB1	166	181	153	178
DGCC7710	99	386	138	231
PrtS^-^	33	948	29	1110
SrtA^-^	115	361	143	213

Kinetics parameters: Vmax, maximum acidification rate in pH units x 10^-4^ per minute and pH 5.2, time to reach pH 5.2 in minutes

Altogether, our results show that the transfer of the *prtS* island between *S. thermophilus* strains is sufficient as such to convert slow acidifying strains into transformants displaying fast activities similar to PrtS^+^ natural isolates. In addition, acquisition of this property was shown to be independent of the *prtS* copy number or the chromosomal integration locus.

## Conclusion

To our knowledge, we performed for the first time a stable and fully functional transfer of the *prtS* locus in PrtS-deficient backgrounds of *S. thermophilus*. By comparing the PrtS^+^ derivative transformants isolated in this study to (i) natural fast and slow acidifying isolates and (ii) *prtS* and *srtA* mutants, we have ultimately shown that PrtS is the most relevant trait responsible for rapid milk acidification. In monoculture, acquisition of a proteolytic system capable of producing short oligopeptides from the casein matrix fulfils thus optimally the nutritional requirements of *S. thermophilus* in milk. The housekeeping sortase A is not required for full PrtS activity and optimal milk acidification. However, we can not exclude that it could be required in other culturing conditions or for the anchorage and activity of others putative sortase substrates. In absence of sortase A, it is probable that most of active PrtS proteins remain anchored in the cell membrane through their C-terminal hydrophobic domain (downstream of the LPXTG motif). Further studies would be required to fully explore the relation between the SrtA protein and its PrtS substrate in *S. thermophilus*.

The PrtS^+^ phenotype was previously reported to be only present in a few strains of *S. thermophilus*. However, since a decade the proportion of strains isolated from industrial dairy products displaying this phenotype has sharply increased, indicating a significant interest of the food industry for this infrequent adaptation [11-15]. The transfer protocol developed in this work is applicable to all transformable *S. thermophilus* strains, provided the insertion sites for *prtS* island i.e. IS*Sth1* or IS*1167* elements are present in their genome. It will thus provide the dairy industry with novel and improved starter strains of *S. thermophilus*, which performed better under fermentation processes and may have a non-Genetically Modified Microorganisms (GMM) status according to the European Legislation [20,30].

## List of abbreviations used

CEP: Cell-Envelope Proteinases; FSDA: Fast Slow Difference Agar; GMM: Genetically Modified Microorganisms; IS: Insertion Sequence; LAB: Lactic Acid Bacteria; ORF: Open Reading Frames; PCR: Polymerase Chain Reaction; WT: Wild type.

## Competing interests

The authors declare that they have no competing interests.

## Authors’ contributions

PHols, LF and DD designed the project; DD, LF, MH performed the experimental work; DD, LF, PHorvath, CF, PHols and PB analysed the data; DD and LF wrote the paper; PHorvath, CF, PHols and PB critically reviewed the paper. All authors approved the final manuscript.

## Supplementary Material

Additional file 1 supplementary material – Table S1, Figs S1-S5. Click here for file
