# Supplementary material for: The fast milk acidifying phenotype of Streptococcus thermophilus can be acquired by natural transformation of the genomic island encoding the cell-envelope proteinase PrtS
Source: Microb Cell Fact. 2011 Aug 30;10(Suppl 1):S21. doi: 10.1186/1475-2859-10-S1-S21 (PMC3231928; doi:10.1186/1475-2859-10-S1-S21)
Supplement: Additional file 1 — supplementary material – Table S1, Figs S1-S5. [file 1475-2859-10-S1-S21-S1.pdf]

Table S1. Primers used in this study

Primer	Sequence (5' to 3')	Target
**Primers used for the construction of *srtA* deletion mutants by natural transformation of overlap PCR products**		
DNdelstrA1	TACATTCCCTTTAGTAACGTGAAGCCTTCAATAAGTCATAC	*srtA*
DNdelstrA2	TAGAGAATAATAAATACCAGCCAAGGCACTTTCCCC	*ster_1253*
UpdelstrA2	CCTTATGGGATTTATCTTCCTTATTTACGATGCTTTTTAGG	*srtA*
UpdelstrA1	AAAACCAGAACGTGTTATTGCTATCATCAAGGAAGAG	*ster_1256*
**Primers used for the construction of *prtS* deletion mutants by natural transformation of overlap PCR products**		
DnDelprtSA	TACATTCCCTTTAGTAACGTGAATTGTTAGCAGCGAAAGC	*prtS*
DnDelprtSB	TGACAATCTCAATTCGTTCTTCAAATGTTGTCTTGCGTCC	IS*Sth1 orf1*
UpDelprtSA	ATTGGGTTGGTGGTTTTGAGTTCCTATGTGATAATG	*eriC*
UpDelprtSB	CCTTATGGGATTTATCTTCCTTAAGTTCCAATTTTATACTTCCG	*prtS*
**Primers used for detection of *prt*S**		
PrtSthF	ATACCTGCACCTTGTTGGCG	*prtS*
PrtSthR	GGTTTCTGTTGTTATTGCAGC	*prtS*
UpDelprtSA	ATTGGGTTGGTGGTTTTGAGTTCCTATGTGATAATG	*eriC*
DnDelprtSB	TGACAATCTCAATTCGTTCTTCAAATGTTGTCTTGCGTCC	IS*Sth1 orf1*
**Primers used for validation of *prtS* insertion obtained by natural transformation**		
EXT62BlpT	ATCCGCTAAAGCAGTTCCTACCG	*blpT*
EXT63BlpR	TCTTTTCCGAAGGTCATAAGTTC	*blpR*
LocPrtSER0850	AAGCATCGATAAAGCCGC	*ster_0850*
PRTSTHF2	ATTGTTGCAGAATTACCACGATATGCCG	*prtS*
seqprtS11	TTTACCTCGTCCAGTCACTTTGCAAGC	*prtS*
STER_0839	TACAGTGGCAGACAATGGCCCTGGTATTGC	*ster_0839*
STER_0843	AATCAACCTGAATTCCTGTCTCGGC	*ster_0843*
STER_0845	TTCCAATTTCACAAATTCATGCC	*ster_0845*
STER_0847	TAGTTTCTCAGAGATAGACTTTGG	*ster_0847*
STER_0850	ATTGGCTAACTAATCGAAAACTCCAGTAGGAG	*ster_0850*
STND0227	TTGGGGAGATAAGGTTGGACAAAAGGGG	*STND0227*
STND0229	ATACCTCAACCAGATGTATGGTGCAGG	*STND0229*
STND0510	TAAGCATCTCCACGAACAGCACGAG	*STND0510*
STND0513	AGGTGCTTCTGAGAAGGCTGCCG	*STND0513*
STND0822	TATGAGTCTACGCCAATTCCGTCC	*STND0822*
STND0825	AGCGAACAAATCTTGACCATCTTCAGAGG	*STND0825*
STND0899	AAAAGATGCGGTCAATCCGATTGTCC	*STND0899*
STND0902	TGATAGTAACCTTACCATGCAGACCCAC	*STND0902*
STND1130	TTGTGCATCTTGACTAGTGGTGTAGCGGC	*STND1130*
STND1132	ATTGTTGCTGGTATTCCAGTTGGTTCTGG	*STND1132*
STND1213	AAGTTCCTCACCATTTGCAGGTGCG	*STND1213*
STND1214	TGGTATGTGTGGTGAAATGGCCGG	*STND1214*
stu0861	TGATTAAACAGAGGTTGAAATCGGGGGC	*stu0861*
stu0868	TAAGGGAGGTTCTGCTCTATTAGTGGGAG	*stu0868*
stu0897	TATCTGTAGTAACGAAGAGTGTGAC	*stu897*
stu0900	TTAGGAAGTATTTCTTCCGGTTATGGG	*stu0900*
stu0900	TTAGGAAGTATTTCTTCCGGTTATGGG	*stu900*
stu1075	GATTAAAAGGCGGTTGAGTTGAGCTAACCC	*stu1075*
stu1089	ATATCCTTGGTATTCGTGGATTTATCGCCC	*stu1089*
stu1354	TTTGGATTTGACATTTTTAGCACTCC	*stu1354*
stu1357	ATCTTGTGTATCCTCTTGTCTCTCG	*stu1357*
stu1833	AAACACCCTCAAATTAAAGATGACC	*stu1833*
stu1836	TTTACACCTAGTGTTGGGATTACAGG	*stu1836*

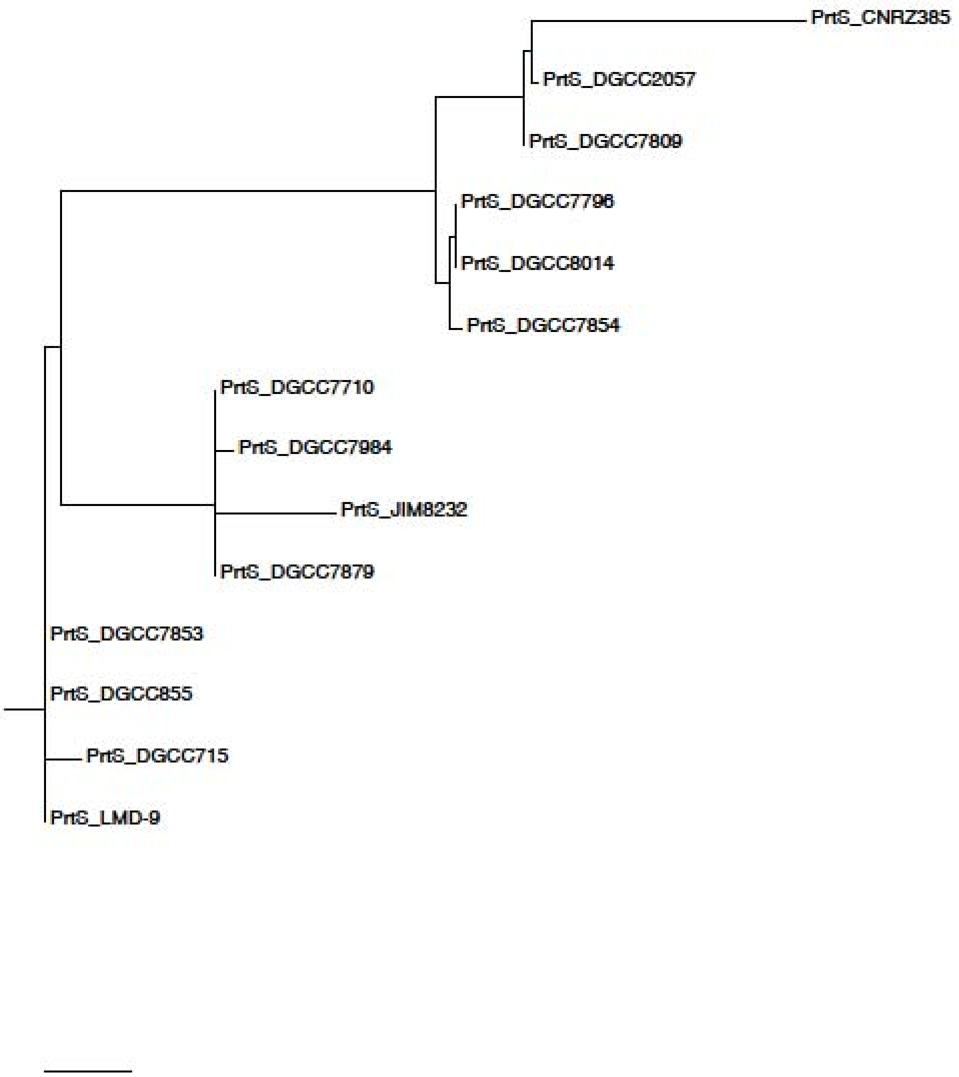

Figure S1. Phylogenetic tree constructed from the amino acid sequences of PrtS proteins from *S. thermophilus*. Sequence alignment and phylogenetic tree (neighbour-joining method) were performed using the ClustalW2 package with default parameters except gap open and gap extension parameters fixed at 100 and 10, respectively (http://www.ebi.ac.uk/Tools/msa/clustalw2/). Genebank accession numbers: *S. thermophilus* LMD-9, ABJ66087; CNRZ385, AF243528; JIM8232, FJ200299.

```
PrtS_DGCC715        MKKKETFSLRKYKIGTVSVLLGAVFLFAGAPSVAADEVTSLVETKVEATVPDVIVSESAS 60
PrtS_LMD-9          MKKKETFSLRKYKIGTVSVLLGAVFLFAGAPSVAADELTSLVETKVEATVPDAIVSESAS 60
PrtS_DGCC855        MKKKETFSLRKYKIGTVSVLLGAVFLFAGAPSVAADELTSLVETKVEATVPDAIVSESAS 60
PrtS_DGCC7853       MKKKETFSLRKYKIGTVSVLLGAVFLFAGAPSVAADELTSLVETKVEATVPDAIVSESAS 60
PrtS_DGCC7984       MKKKETFSLRKYKIGTVSVLLGAVFLFAGAPSVAADELTSLVETKVEATVPDAIVSESAS 60
PrtS_JIM8232        MKKKETFSLRKYKIGTVSVLLGAVFLFAGAPSVAADELTSLVETKVEATVPDAIVSESAS 60
PrtS_DGCC7710       MKKKETFSLRKYKIGTVSVLLGAVFLFAGAPSVAADELTSLVETKVEATVPDAIVSESAS 60
PrtS_DGCC7879       MKKKETFSLRKYKIGTVSVLLGAVFLFAGAPSVAADELTSLVETKVEATVPDAIVSESAS 60
PrtS_CNRZ385        MKKKETFSLRKYKIGTVSVLLGAVFLFAGAPSVAADELTSLVETKVEATVPDAIVSESAS 60
PrtS_DGCC2057       MKKKETFSLRKYKIGTVSVLLGAVFLFAGAPSVAADELTSLVETKVEATVPDAIVSESAS 60
PrtS_DGCC7809       MKKKETFSLRKYKIGTVSVLLGAVFLFAGAPSVAADELTSLVETKVEATVPDAIVSESAS 60
PrtS_DGCC7796       MKKKETFSLRKYKIGTVSVLLGAVFLFAGAPSVAADELTSLVETKVEATVPDAIVSESAS 60
PrtS_DGCC8014       MKKKETFSLRKYKIGTVSVLLGAVFLFAGAPSVAADELTSLVETKVEATVPDAIVSESAS 60
PrtS_DGCC7854       MKKKETFSLRKYKIGTVSVLLGAVFLFAGAPSVAADELTSLVETKVEATVPDAIVSESAS 60
                    *************************************:**************.*******

PrtS_DGCC715        ES--------------------------------PVVEELVDTSVEATPTDVTTTDNVEE 88
PrtS_LMD-9          ESPVAEELVDTSVEATSTDVTTTDNEEETLGSESPVVEELVDTSVEATPTDVTTTDNVEE 120
PrtS_DGCC855        ESPVAEELVDTSVEATSTDVTTTDNEEETLGSESPVVEELVDTSVEATPTDVTTTDNVEE 120
PrtS_DGCC7853       ESPVAEELVDTSVEATSTDVTTTDNEEETLGSESPVVEELVDTSVEATPTDVTTTDNVEE 120
PrtS_DGCC7984       ESPVAEELVDTSVEATSTDVTTTDNEEETLGSESPVVEELVDTSVEATSTDVTTTDNVEE 120
PrtS_JIM8232        ESPVAEELVDTSVEATSTDVTTTDNEEETLGSESPVVEELVDTSVEATSTDVTTTDNVEE 120
PrtS_DGCC7710       ESPVAEELVDTSVEATSTDVTTTDNEEETLGSESPVVEELVDTSVEATSTDVTTTDNVEE 120
PrtS_DGCC7879       ESPVAEELVDTSVEATSTDVTTTDNEEETLGSESPVVEELVDTSVEATSTDVTTTDNVEE 120
PrtS_CNRZ385        ES--------------------------------PVVEELVDTSVEATSTDVTTTDNEEE 88
PrtS_DGCC2057       ESPVAEELVDTSVEATPTDVTTTDNEEETLGSESPVVEELVDTSVEATSTDVTTTDNEEE 120
PrtS_DGCC7809       ESPVAEELVDTSVEATSTDVTTTDNEEETLGSESPVVEELVDTSVEATSTDVTTTDNEEE 120
PrtS_DGCC7796       ESPVAEELVDTSVEATSTDVTTTDNEEETLGSESPVVEELVDTSVEATSTDVTTTDNEEE 120
PrtS_DGCC8014       ESPVAEELVDTSVEATSTDVTTTDNEEETLGSESPVVEELVDTSVEATSTDVTTTDNEEE 120
PrtS_DGCC7854       ESPVAEELVDTSVEATSTDVTTTDNEE--------------------------------E 88
                    **                                                         *

PrtS_DGCC715        TLGSEALENITNTEVEATQPAVETPAISEKKVEEEEKLSVADETTAITNQEEAKPQNIDS 148
PrtS_LMD-9          TLGSEALENITNTEVEATQPAVETPAISEKKVEEEEKLSVADETTAITNQEEAKPQNIDS 180
PrtS_DGCC855        TLGSEALENITNTEVEATQPAVETPAISEKKVEEEEKLSVADETTAITNQEEAKPQNIDS 180
PrtS_DGCC7853       TLGSEALENITNTEVEATQPAVETPAISEKKVEEEEKLSVADETTAITNQEEAKPQNIDS 180
PrtS_DGCC7984       TLGSEALENITNTEVEATQPAVETPAISEKKVEEEEKLSVADETTAITNQEEAKPQNIDS 180
PrtS_JIM8232        TLGSEALENITNTEVEATQPAVETPAISEKKVEEEEKLSVADETTAITNQEEAKPQNIDS 180
PrtS_DGCC7710       TLGSEALENITNTEVEATQPAVETPAISEKKVEEEEKLSVADETTAITNQEEAKPQNIDS 180
PrtS_DGCC7879       TLGSEALENITNTEVEATQPAVETPAISEKKVEEEEKLSVADETTAITNQEEAKPQNIDS 180
PrtS_CNRZ385        TPGSEALENSANTEVETTQPAVETPAISEKKVEEEEKLSVADETTAITNQEEAKPQNIDS 148
PrtS_DGCC2057       TPGSEALENSANTEVETTQPAVETPAISEKKVEEEEKLSVADETTAITNQEEAKPQNIDS 180
PrtS_DGCC7809       TPGSEALENSANTEVETTQPAVETPAISEKKVEEEEKLSVADETTAITNQEEAKPQNIDS 180
PrtS_DGCC7796       TPGSEALENSANTEVETTQPAVETPAISEKKVEEEEKLSVADETTAITNQEEAKPQNIDS 180
PrtS_DGCC8014       TPGSEALENSANTEVETTQPAVETPAISEKKVEEEEKLSVADETTAITNQEEAKPQNIDS 180
PrtS_DGCC7854       TPGSEALENSANTEVETTQPAVETPAISEKKVEEEEKLSVADETTAITNQEEAKPQNIDS 148
                    * ******* :*****:*******************************************

PrtS_DGCC715        NTIITVPKVWDSGYKGEGTVVAIIDSGLDVDHDVLHISDLSTAKYKSEKEIEAAKEVAGI 208
PrtS_LMD-9          NTIITVPKVWDSGYKGEGTVVAIIDSGLDVDHDVLHISDLSTAKYKSEKEIEAAKEVAGI 240
PrtS_DGCC855        NTIITVPKVWDSGYKGEGTVVAIIDSGLDVDHDVLHISDLSTAKYKSEKEIEAAKEVAGI 240
PrtS_DGCC7853       NTIITVPKVWDSGYKGEGTVVAIIDSGLDVDHDVLHISDLSTAKYKSEKEIEAAKEVAGI 240
PrtS_DGCC7984       NTIITVPKVWDSGYKGEGTVVAIIDSGLDVDHDVLHISDLSTAKYKSEKEIEAAKEVAGI 240
PrtS_JIM8232        NTIITVPKVWDSGYKGEGTVVAIIDSGLDVDHDVLHISDLSTAKYKSEKEIEAAKEVAGI 240
PrtS_DGCC7710       NTIITVPKVWDSGYKGEGTVVAIIDSGLDVDHDVLHISDLSTAKYKSEKEIEAAKEVAGI 240
PrtS_DGCC7879       NTIITVPKVWDSGYKGEGTVVAIIDSGLDVDHDVLHISDLSTAKYKSEKEIEAAKEVAGI 240
PrtS_CNRZ385        NTIITVPKVWYSGYKGEGTVVAIIDSGLDVDHDVLHISDLSTAKYKSEKEIEAAKEAAGI 208
PrtS_DGCC2057       NTIITVPKVWYSGYKGEGTVVAIIDSGLDVDHDVLHISDLSTAKYKSEKEIEAAKEAAGI 240
PrtS_DGCC7809       NTIITVPKVWYSGYKGEGTVVAIIDSGLDVDHDVLHISDLSTAKYKSEKEIEAAKEAAGI 240
PrtS_DGCC7796       NTIITVPKVWYSGYKGEGTVVAIIDSGLDVDHDVLHISDLSTAKYKSEKEIEAAKEAAGI 240
PrtS_DGCC8014       NTIITVPKVWYSGYKGEGTVVAIIDSGLDVDHDVLHISDLSTAKYKSEKEIEAAKEAAGI 240
PrtS_DGCC7854       NTIITVPKVWYSGYKGEGTVVAIIDSGLDVDHDVLHISDLSTAKYKSEKEIEAAKEAAGI 208
                    ********** **********************************************.***

PrtS_DGCC715        SYGEWFNDKVVFGYNYVDVNTVLKEEDKRSHGMHVTSIATGNPTQPVAGQLMYGVAPEAQ 268
PrtS_LMD-9          SYGEWFNDKVVFGYNYVDVNTVLKEEDKRSHGMHVTSIATGNPTQPVAGQLMYGVAPEAQ 300
PrtS_DGCC855        SYGEWFNDKVVFGYNYVDVNTVLKEEDKRSHGMHVTSIATGNPTQPVAGQLMYGVAPEAQ 300
PrtS_DGCC7853       SYGEWFNDKVVFGYNYVDVNTVLKEEDKRSHGMHVTSIATGNPTQPVAGQLMYGVAPEAQ 300
```

```
PrtS_DGCC7984      SYGEWFNDKVVFGYNYVDVNTVLKEEDKRSHGMHVTSIATGNPTQPVAGQLMYGVAPEAQ 300
PrtS_JIM8232       SYGEWFNDKVVFGYNYVDVNTVLKEEDKRSHGMHVTSIATGNPTQPVAGQLMYGVAPEAQ 300
PrtS_DGCC7710      SYGEWFNDKVVFGYNYVDVNTVLKEEDKRSHGMHVTSIATGNPTQPVAGQLMYGVAPEAQ 300
PrtS_DGCC7879      SYGEWFNDKVVFGYNYVDVNTVLKEEDKRSHGMHVTSIATGNPTQPVAGQLMYGVAPEAQ 300
PrtS_CNRZ385       TYGEWFNDKVVFGYNYVDVNTVLKEEDKRSHGMHVTSIATGNPTQPVAGQLMYGVAPEAQ 268
PrtS_DGCC2057      TYGEWFNDKVVFGYNYVDVNTVLKEEDKRSHGMHVTSIATGNPTQPVAGQLMYGVAPEAQ 300
PrtS_DGCC7809      TYGEWFNDKVVFGYNYVDVNTVLKEEDKRSHGMHVTSIATGNPTQPVAGQLMYGVAPEAQ 300
PrtS_DGCC7796      TYGEWFNDKVVFGYNYVDVNTVLKEEDKRSHGMHVTSIATGNPTQPVAGQLMYGVAPEAQ 300
PrtS_DGCC8014      TYGEWFNDKVVFGYNYVDVNTVLKEEDKRSHGMHVTSIATGNPTQPVAGQLMYGVAPEAQ 300
PrtS_DGCC7854      TYGEWFNDKVVFGYNYVDVNTVLKEEDKRSHGMHVTSIATGNPTQPVAGQLMYGVAPEAQ 268
                   :***********************************************************

PrtS_DGCC715       VMFMRVFSDLKATTGAALYVKAIEDAVKLGADSINLSLGGANGSVVNMNENVTAAIEAAR 328
PrtS_LMD-9         VMFMRVFSDLKATTGAALYVKAIEDAVKLGADSINLSLGGANGSVVNMNENVTAAIEAAR 360
PrtS_DGCC855       VMFMRVFSDLKATTGAALYVKAIEDAVKLGADSINLSLGGANGSVVNMNENVTAAIEAAR 360
PrtS_DGCC7853      VMFMRVFSDLKATTGAALYVKAIEDAVKLGADSINLSLGGANGSVVNMNENVTAAIEAAR 360
PrtS_DGCC7984      VMFMRVFSDLKATTGAALYVKAIEDAVKLGADSINLSLGGANGSVVNMNENVTAAIEAAR 360
PrtS_JIM8232       VMFMRVFSDLKATTGAALYVKAIEDAVKLGADSINLSLGGANGSVVNMNENVTAAIEAAR 360
PrtS_DGCC7710      VMFMRVFSDLKATTGAALYVKAIEDAVKLGADSINLSLGGANGSVVNMNENVTAAIEAAR 360
PrtS_DGCC7879      VMFMRVFSDLKATTGAALYVKAIEDAVKLGADSINLSLGGANGSVVNMNENVTAAIEAAR 360
PrtS_CNRZ385       VMFMRVFSDLKATTGAALYVKAIEDAVKLGADSINLSLGGANGSVVNMNENVTAAIEAAR 328
PrtS_DGCC2057      VMFMRVFSDLKATTGAALYVKAIEDAVKLGADSINLSLGGANGSVVNMNENVTAAIEAAR 360
PrtS_DGCC7809      VMFMRVFSDLKATTGAALYVKAIEDAVKLGADSINLSLGGANGSVVNMNENVTAAIEAAR 360
PrtS_DGCC7796      VMFMRVFSDLKATTGAALYVKAIEDAVKLGADSINLSLGGANGSVVNMNENVTAAIEAAR 360
PrtS_DGCC8014      VMFMRVFSDLKATTGAALYVKAIEDAVKLGADSINLSLGGANGSVVNMNENVTAAIEAAR 360
PrtS_DGCC7854      VMFMRVFSDLKATTGAALYVKAIEDAVKLGADSINLSLGGANGSVVNMNENVTAAIEAAR 328
                   ************************************************************

PrtS_DGCC715       RAGVSVVIAAGNDGTFGSGHSNPSADYPDYGLVGAPSTARDAISVASYNNTTVGSKVINI 388
PrtS_LMD-9         RAGVSVVIAAGNDGTFGSGHSNPSADYPDYGLVGAPSTARDAISVASYNNTTVGSKVINI 420
PrtS_DGCC855       RAGVSVVIAAGNDGTFGSGHSNPSADYPDYGLVGAPSTARDAISVASYNNTTVGSKVINI 420
PrtS_DGCC7853      RAGVSVVIAAGNDGTFGSGHSNPSADYPDYGLVGAPSTARDAISVASYNNTTVGSKVINI 420
PrtS_DGCC7984      RAGVSVVIAAGNDGTFGSGHSNPSADYPDYGLVGAPSTARDAISVASYNNTTVGSKVINI 420
PrtS_JIM8232       RAGVSVVIAAGNDGTFGSGHSNPSADYPDYGLVGAPSTARDAISVASYNNTTVGSKVINI 420
PrtS_DGCC7710      RAGVSVVIAAGNDGTFGSGHSNPSADYPDYGLVGAPSTARDAISVASYNNTTVGSKVINI 420
PrtS_DGCC7879      RAGVSVVIAAGNDGTFGSGHSNPSADYPDYGLVGAPSTARDAISVASYNNTTVGSKVINI 420
PrtS_CNRZ385       RAGVSVVIAAGNDGTFGSGHSNPSADYPDYGLVGAPSTAHDAISVASYNNTTVGSKVINI 388
PrtS_DGCC2057      RAGVSVVIAAGNDGTFGSGHSNPSADYPDYGLVGAPSTAHDAISVASYNNTTVGSKVINI 420
PrtS_DGCC7809      RAGVSVVIAAGNDGTFGSGHSNPSADYPDYGLVGAPSTAHDAISVASYNNTTVGSKVINI 420
PrtS_DGCC7796      RAGVSVVIAAGNDGTFGSGHSNPSADYPDYGLVGAPSTAHDAISVASYNNTTVGSKVINI 420
PrtS_DGCC8014      RAGVSVVIAAGNDGTFGSGHSNPSADYPDYGLVGAPSTAHDAISVASYNNTTVGSKVINI 420
PrtS_DGCC7854      RAGVSVVIAAGNDGTFGSGHSNPSADYPDYGLVGAPSTAHDAISVASYNNTTVGSKVINI 388
                   ***************************************:********************

PrtS_DGCC715       IGLENNADLNYGKSSFDNPEKSPVPFEIGKEYEYVYAGIGQASDFDGLDLTGKLALIKRG 448
PrtS_LMD-9         IGLENNADLNYGKSSFDNPEKSPVPFEIGKEYEYVYAGIGQASDFDGLDLTGKLALIKRG 480
PrtS_DGCC855       IGLENNADLNYGKSSFDNPEKSPVPFEIGKEYEYVYAGIGQASDFDGLDLTGKLALIKRG 480
PrtS_DGCC7853      IGLENNADLNYGKSSFDNPEKSPVPFEIGKEYEYVYAGIGQASDFDGLDLTGKLALIKRG 480
PrtS_DGCC7984      IGLENNADLNYGKSSFDNPEKSPVPFEIGKEYEYVYAGIGQASDFDGLDLTGKLALIKRG 480
PrtS_JIM8232       IGLENNADLNYGKSSFDNPEKSPVPFEIGKEYEYVYAGIGQASDFDGLDLTGKLALIKRG 480
PrtS_DGCC7710      IGLENNADLNYGKSSFDNPEKSPVPFEIGKEYEYVYAGIGQASDFDGLDLTGKLALIKRG 480
PrtS_DGCC7879      IGLENNADLNYGKSSFDNPEKSPVPFEIGKEYEYVYAGIGQASDFDGLDLTGKLALIKRG 480
PrtS_CNRZ385       IGLENNADLNYGKSSFDNPEKSPVPFEIGKEYEYVYAGIGQASDFDGLDLTGKLALIKRG 448
PrtS_DGCC2057      IGLENNADLNYGKSSFDNPEKSPVPFEIGKEYEYVYAGIGQASDFDGLDLTGKLALIKRG 480
PrtS_DGCC7809      IGLENNADLNYGKSSFDNPEKSPVPFEIGKEYEYVYAGIGQASDFDGLDLTGKLALIKRG 480
PrtS_DGCC7796      IGLENNADLNYGKSSFDNPEKSPVPFEIGKEYEYVYAGIGQASDFDGLDLTGKLALIKRG 480
PrtS_DGCC8014      IGLENNADLNYGKSSFDNPEKSPVPFEIGKEYEYVYAGIGQASDFDGLDLTGKLALIKRG 480
PrtS_DGCC7854      IGLENNADLNYGKSSFDNPEKSPVPFEIGKEYEYVYAGIGQASDFDGLDLTGKLALIKRG 448
                   ************************************************************

PrtS_DGCC715       TISFSEKIANATAAGAVGVVIFNSRPDEANVSMQLDDTAIAIPSVFIPLEFGEALAANSY 508
PrtS_LMD-9         TISFSEKIANATAAGAVGVVIFNSRPDEANVSMQLDDTAIAIPSVFIPLEFGEALAANSY 540
PrtS_DGCC855       TISFSEKIANATAAGAVGVVIFNSRPDEANVSMQLDDTAIAIPSVFIPLEFGEALAANSY 540
PrtS_DGCC7853      TISFSEKIANATAAGAVGVVIFNSRPDEANVSMQLDDTAIAIPSVFIPLEFGEALAANSY 540
PrtS_DGCC7984      TISFSEKIANATAAGAVGVVIFNSRPDEANVSMQLDDTAIAIPSVFIPLEFGEALAANSY 540
PrtS_JIM8232       TISFSEKIANATAAGAVGVVIFNSRPDEANVSMQLDDTAIAIPSVFIPLEFGEALAANSY 540
PrtS_DGCC7710      TISFSEKIANATAAGAVGVVIFNSRPDEANVSMQLDDTAIAIPSVFIPLEFGEALAANSY 540
PrtS_DGCC7879      TISFSEKIANATAAGAVGVVIFNSRPDEANVSMQLDDTAIAIPSVFIPLEFGEALAANSY 540
```

```
PrtS_CNRZ385       TITFSEKIANATAAGAVGVVIFNSRPGEANVSMQLDDTAIAIPSVFIPLEFGEALAANSY 508
PrtS_DGCC2057      TITFSEKIANATAAGAVGVVIFNSRPGEANVSMQLDDTAIAIPSVFIPLEFGEALAANSY 540
PrtS_DGCC7809      TITFSEKIANATAAGAVGVVIFNSRPGEANVSMQLDDTAIAIPSVFIPLEFGEALAANSY 540
PrtS_DGCC7796      TITFSEKIANATAAGAVGVVIFNSRPGEANVSMQLDDTAIAIPSVFIPLEFGEALAANSY 540
PrtS_DGCC8014      TITFSEKIANATAAGAVGVVIFNSRPGEANVSMQLDDTAIAIPSVFIPLEFGEALAANSY 540
PrtS_DGCC7854      TITFSEKIANATAAGAVGVVIFNSRPGEANVSMQLDDTAIAIPSVFIPLEFGEALAANSY 508
                   **:***********************.*********************************

PrtS_DGCC715       KIAFNNETDIRPNPEAGLLSDFSSWGLSADGELKPDLAAPGGAIYAAINDNDYANMQGTS 568
PrtS_LMD-9         KIAFNNETDIRPNPEAGLLSDFSSWGLSADGELKPDLAAPGGAIYAAINDNDYANMQGTS 600
PrtS_DGCC855       KIAFNNETDIRPNPEAGLLSDFSSWGLSADGELKPDLAAPGGAIYAAINDNDYANMQGTS 600
PrtS_DGCC7853      KIAFNNETDIRPNPEAGLLSDFSSWGLSADGELKPDLAAPGGAIYAAINDNDYANMQGTS 600
PrtS_DGCC7984      KIAFNNETDIRPNPEAGLLSDFSSWGLSADGELKPDLAAPGGAIYAAINDNDYANMQGTS 600
PrtS_JIM8232       KIAFNNETDIRPNPEAGLLSDFSSWGLSADGELKPDLAAPGGAIYAAINDNDYANMQGTS 600
PrtS_DGCC7710      KIAFNNETDIRPNPEAGLLSDFSSWGLSADGELKPDLAAPGGAIYAAINDNDYANMQGTS 600
PrtS_DGCC7879      KIAFNNETDIRPNPEAGLLSDFSSWGLSADGELKPDLAAPGGAIYAAINDNDYANMQGTS 600
PrtS_CNRZ385       KIAFNNETDIRPNPEAGLLSDFSSWGLSADGELKPDLAAPGGAIYAAINDNDYANMQGTS 568
PrtS_DGCC2057      KIAFNNETDIRPNPEAGLLSDFSSWGLSADGELKPDLAAPGGAIYAAINDNDYANMQGTS 600
PrtS_DGCC7809      KIAFNNETDIRPNPEAGLLSDFSSWGLSADGELKPDLAAPGGAIYAAINDNDYANMQGTS 600
PrtS_DGCC7796      KIAFNNETDIRPNPEAGLLSDFSSWGLSADGELKPDLAAPGGAIYAAINDNDYANMQGTS 600
PrtS_DGCC8014      KIAFNNETDIRPNPEAGLLSDFSSWGLSADGELKPDLAAPGGAIYAAINDNDYANMQGTS 600
PrtS_DGCC7854      KIAFNNETDIRPNPEAGLLSDFSSWGLSADGELKPDLAAPGGAIYAAINDNDYANMQGTS 568
                   ************************************************************

PrtS_DGCC715       MASPHVAGAAVLVKQYLQATYPTKSPQEIEALVKHLLMSTAKAHVNKETTAYTSPRQQGA 628
PrtS_LMD-9         MASPHVAGAAVLVKQYLQATYPTKSPQEIEALVKHLLMSTAKAHVNKETTAYTSPRQQGA 660
PrtS_DGCC855       MASPHVAGAAVLVKQYLQATYPTKSPQEIEALVKHLLMSTAKAHVNKETTAYTSPRQQGA 660
PrtS_DGCC7853      MASPHVAGAAVLVKQYLQATYPTKSPQEIEALVKHLLMSTAKAHVNKETTAYTSPRQQGA 660
PrtS_DGCC7984      MASPHVAGAAVLVKQYLQATYPTKSPQEIEALVKHLLMSTAKAHVNKETTAYTSPRQQGA 660
PrtS_JIM8232       MASPHVAGAAVLVKQYLQATYPTKSPQEIEALVKHLLMSTAKAHVNKETTAYTSPRQQGA 660
PrtS_DGCC7710      MASPHVAGAAVLVKQYLQATYPTKSPQEIEALVKHLLMSTAKAHVNKETTAYTSPRQQGA 660
PrtS_DGCC7879      MASPHVAGAAVLVKQYLQATYPTKSPQEIEALVKHLLMSTAKAHVNKETTAYTSPRQQGA 660
PrtS_CNRZ385       MASPHVAGAAVLVKQYLLATYPTKSPQEIEALVKHLLMSTAKAHVNKETTAYTSPRQQGA 628
PrtS_DGCC2057      MASPHVAGAAVLVKQYLLATYPTKSPQEIEALVKHLLMSTAKAHVNKETTAYTSPRQQGA 660
PrtS_DGCC7809      MASPHVAGAAVLVKQYLLATYPTKSPQEIEALVKHLLMSTAKAHVNKETTAYTSPRQQGA 660
PrtS_DGCC7796      MASPHVAGAAVLVKQYLLATYPTKSPQEIEALVKHLLMSTAKAHVNKETTAYTSPRQQGA 660
PrtS_DGCC8014      MASPHVAGAAVLVKQYLLATYPTKSPQEIEALVKHLLMSTAKAHVNKETTAYTSPRQQGA 660
PrtS_DGCC7854      MASPHVAGAAVLVKQYLLATYPTKSPQEIEALVKHLLMSTAKAHVNKETTAYTSPRQQGA 628
                   ***************** ******************************************

PrtS_DGCC715       GIIDTAAAISTGLYLTGEDGYGSITLGNVEDTFSFTVTLHNITNEDKTLNYSTQLTTDTV 688
PrtS_LMD-9         GIIDTAAAISTGLYLTGEDGYGSITLGNVEDTFSFTVTLHNITNEDKTLNYSTQLTTDTV 720
PrtS_DGCC855       GIIDTAAAISTGLYLTGEDGYGSITLGNVEDTFSFTVTLHNITNEDKTLNYSTQLTTDTV 720
PrtS_DGCC7853      GIIDTAAAISTGLYLTGEDGYGSITLGNVEDTFSFTVTLHNITNEDKTLNYSTQLTTDTV 720
PrtS_DGCC7984      GIIDTAAAISTGLYLTGEDGYGSITLGNVEDIFSFTVTLHNITNEDKTLNYSTQLTTDTV 720
PrtS_JIM8232       GIIDTAAAISTGLYLTGEDGYGSITLGNVEDTFSFTVTLHNITNEDKTLNYSTQLTTDTV 720
PrtS_DGCC7710      GIIDTAAAISTGLYLTGEDGYGSITLGNVEDTFSFTVTLHNITNEDKTLNYSTQLTTDTV 720
PrtS_DGCC7879      GIIDTAAAISTGLYLTGEDGYGSITLGNVEDTFSFTVTLHNITNEDKTLNYSTQLTTDTV 720
PrtS_CNRZ385       GIIDTAAAISTGLYLTGEDGYGSITLGNVEDTFSFTVTLHNITNEDKTLNYSTQLTTDTA 688
PrtS_DGCC2057      GIIDTAAAISTGLYLTGEDGYGSITLGNVEDTFSFTVTLHNITNEDKTLNYSTQLTTDTA 720
PrtS_DGCC7809      GIIDTAAAISTGLYLTGEDGYGSITLGNVEDTFSFTVTLHNITNEDKTLNYSTQLTTDTA 720
PrtS_DGCC7796      GIIDTAAAISTGLYLTGEDGYGSITLGNVEDTFSFTVTLHNITNEDKTLNYSTQLTTDTA 720
PrtS_DGCC8014      GIIDTAAAISTGLYLTGEDGYGSITLGNVEDTFSFTVTLHNITNEDKTLNYSTQLTTDTA 720
PrtS_DGCC7854      GIIDTAAAISTGLYLTGEDGYGSITLGNVEDTFSFTVTLHNITNEDKTLNYSTQLTTDTA 688
                   ******************************* ***************************.

PrtS_DGCC715       QNGLITLAPRLLAEIPGGKVTVQANSSTTVTINVDASSFAEELTGLMKNGYYLEGFVRFT 748
PrtS_LMD-9         QNGLITLAPRLLAEIPGGKVTVQANSSTTVTINVDASSFAEELTGLMKNGYYLEGFVRFT 780
PrtS_DGCC855       QNGLITLAPRLLAEIPGGKVTVQANSSTTVTINVDASSFAEELTGLMKNGYYLEGFVRFT 780
PrtS_DGCC7853      QNGLITLAPRLLAEIPGGKVTVQANSSTTVTINVDASSFAEELTGLMKNGYYLEGFVRFT 780
PrtS_DGCC7984      QNGLITLAPRLLAEIPGGKVTVQANSSTTVTINVDASSFAEELTGLMKNGYYLEGFVRFT 780
PrtS_JIM8232       QNGLITLAPRLLAEIPGGKVTVQANSSTTVTINVDASSFAKELTGLMKNGYYLEGFVRFT 780
PrtS_DGCC7710      QNGLITLAPRLLAEIPGGKVTVQANSSTTVTINVDASSFAEELTGLMKNGYYLEGFVRFT 780
PrtS_DGCC7879      QNGLITLAPRLLAEIPGGKVTVQANSSTTVTINVDASSFAEELTGLMKNGYYLEGFVRFT 780
PrtS_CNRZ385       QKRIDHLGSTSISRDSWRKVTVKANSSTTVTINVDASSFAEELTGLMKNGYYLEGFVRFT 748
PrtS_DGCC2057      QNGLITLAPRLLAEIPGGKVTVKANSSTTVTINVDASSFAEELTGLMKNGYYLEGFVRFT 780
PrtS_DGCC7809      QNGLITLAPRLLAEIPGGKVTVKANSSTTVTINVDASSFAEELTGLMKNGYYLEGFVRFT 780
PrtS_DGCC7796      QNGLITLAPRLLAEIPGGKVTVKANSSTTVTINVDASSFAEELTGLMKNGYYLEGFVRFT 780
```

```
PrtS_DGCC8014      QNGLITLAPRLLAEIPGGKVTVKANSSTTVTINVDASSFAEELTGLMKNGYYLEGFVRFT 780
PrtS_DGCC7854      QNGLITLAPRLLAEIPGGKVTVKANSSTTVTINVDASSFAEELTGLMKNGYYLEGFVRFT 748
                   *: :  *..  ::. .  ****:*****************:*******************

PrtS_DGCC715       DVADGGDIVSIPYIGFRGEFQNLAVLEEPIYNLIADGKGGFYFEPVTAQPDSVDISHHYT 808
PrtS_LMD-9         DVADGGDIVSIPYIGFRGEFQNLAVLEEPIYNLIADGKGGFYFEPVTAQPDSVDISHHYT 840
PrtS_DGCC855       DVADGGDIVSIPYIGFRGEFQNLAVLEEPIYNLIADGKGGFYFEPVTAQPDSVDISHHYT 840
PrtS_DGCC7853      DVADGGDIVSIPYIGFRGEFQNLAVLEEPIYNLIADGKGGFYFEPVTAQPDSVDISHHYT 840
PrtS_DGCC7984      DVADGGDIVSIPYIGFRGEFQNLAVLEEPIYNLIADGKGGFYFEPVTAQPDSVDISHHYT 840
PrtS_JIM8232       DVADGGDIVSIPYIGFRGEFQNLAVLEEPIYNLIADGKGGFYFEPVTAQPDSVDISHHYT 840
PrtS_DGCC7710      DVADGGDIVSIPYIGFRGEFQNLAVLEEPIYNLIADGKGGFYFEPVTAQPDSVDISHHYT 840
PrtS_DGCC7879      DVADGGDIVSIPYIGFRGEFQNLAVLEEPIYNLIADGKGGFYFEPVTAQPDSVDISHHYT 840
PrtS_CNRZ385       DVADDGDIVSIPYVGFRGEFQNLAVLEEPIYNLIADGKGGFYFEPVTAQPNTVDISHHYT 808
PrtS_DGCC2057      DVADDGDIVSIPYVGFRGEFQNLAVLEEPIYNLIADGKGGFYFEPVTAQPNTVDISHHYT 840
PrtS_DGCC7809      DVADDGDIVSIPYVGFRGEFQNLAVLEEPIYNLIADGKGGFYFEPVTAQPNTVDISHHYT 840
PrtS_DGCC7796      DVADDGDIVSIPYVGFRGEFQNLAVLEEPIYNLIADGKGGFYFEPVTAQPNTVDISHHYT 840
PrtS_DGCC8014      DVADDGDIVSIPYVGFRGEFQNLAVLEEPIYNLIADGKGGFYFEPVTAQPNTVDISHHYT 840
PrtS_DGCC7854      DVADDGDIVSIPYVGFRGEFQNLAVLEEPIYNLIADGKGGFYFEPVTAQPNTVDISHHYT 808
                   ****.********:************************************::********

PrtS_DGCC715       GLVTGSTELIYSTDKRSDFAIKKTLGTFKNEAGYFVLELDESGKPHLAISPNGDDNQDSL 868
PrtS_LMD-9         GLVTGSTELIYSTDKRSDFAIKKTLGTFKNEAGYFVLELDESGKPHLAISPNGDDNQDSL 900
PrtS_DGCC855       GLVTGSTELIYSTDKRSDFAIKKTLGTFKNEAGYFVLELDESGKPHLAISPNGDDNQDSL 900
PrtS_DGCC7853      GLVTGSTELIYSTDKRSDFAIKKTLGTFKNEAGYFVLELDESGKPHLAISPNGDDNQDSL 900
PrtS_DGCC7984      GLVTGSTELIYSTDKRSDFAIKKTLGTFKNEAGYFVLELDESGKPHLAISPNGDDNQDSL 900
PrtS_JIM8232       GLVTGSTELIYSTDKRSDFAIKKTLGTFKNEAGYFVLELDESGKPHLAISPNGDDNQDSL 900
PrtS_DGCC7710      GLVTGSTELIYSTDKRSDFAIKKTLGTFKNEAGYFVLELDESGKPHLAISPNGDDNQDSL 900
PrtS_DGCC7879      GLVTGSTELIYSTDKRSDFAIKKTLGTFKNEAGYFVLELDESGKPHLAISPNGDDNQDSL 900
PrtS_CNRZ385       GLVTGSTELIYSTDKRSDSAIKTLG-TFKNKAGYFVLELDESGKPHLAISPNGDDNQDSL 867
PrtS_DGCC2057      GLVTGSTELIYSTDKRSDSAIKTLG-TFKNKAGYFVLELDESGKPHLAISPNGDDNQDSL 899
PrtS_DGCC7809      GLVTGSTELIYSTDKRSDSAIKTLG-TFKNKAGYFVLELDESGKPHLAISPNGDDNQDSL 899
PrtS_DGCC7796      GLVTGSTELIYSTDKRSDSAIKTLG-TFKNKAGYFVLELDESGKPHLAISPNGDDNQDSL 899
PrtS_DGCC8014      GLVTGSTELIYSTDKRSDSAIKTLG-TFKNKAGYFVLELDESGKPHLAISPNGDDNQDSL 899
PrtS_DGCC7854      GLVTGSTELIYSTDKRSDSAIKTLG-TFKNKAGYFVLELDESGKPHLAISPNGDDNQDSL 867
                   ****************** ***.   ****:*****************************

PrtS_DGCC715       AFKGVFLRNYTDLVASVYAADDTERTNPLWESQPQSGNKNFYSGDPKNPKSSIIYPTEWN 928
PrtS_LMD-9         AFKGVFLRNYTDLVASVYAADDTERTNPLWESQPQSGNKNFYSGDPKNPKSSIIYPTEWN 960
PrtS_DGCC855       AFKGVFLRNYTDLVASVYAADDTERTNPLWESQPQSGNKNFYSGDPKNPKSSIIYPTEWN 960
PrtS_DGCC7853      AFKGVFLRNYTDLVASVYAADDTERTNPLWESQPQSGNKNFYSGDPKNPKSSIIYPTEWN 960
PrtS_DGCC7984      AFKGVFLRNYTDLVASVYAADDTERTNPLWESQPQSGNKNFYSGDPKNPKSSIIYPTEWN 960
PrtS_JIM8232       AFKGVFLRNYTDLVASVYAADDTERTNPLWESQPQSGNKNFYSGDPKNPKSSIIYPTEWN 960
PrtS_DGCC7710      AFKGVFLRNYTDLVASVYAADDTERTNPLWESQPQSGNKNFYSGDPKNPKSSIIYPTEWN 960
PrtS_DGCC7879      AFKGVFLRNYTDLVASVYAADDTERTNPLWESQPQSGNKNFYSGDPKNPKSSIIYPTEWN 960
PrtS_CNRZ385       VFKGVFLRNYTDLVASVYAADDTERTNPLWESQPQSGDKNIYSGNPKNPKSSIIYPTEWN 927
PrtS_DGCC2057      VFKGVFLRNYTDLVASVYAADDTERTNPLWESQPQSGDKNIYSGNPKNPKSSIIYPTEWN 959
PrtS_DGCC7809      VFKGVFLRNYTDLVASVYAADDTERTNPLWESQPQSGDKNIYSGNPKNPKSSIIYPTEWN 959
PrtS_DGCC7796      VFKGVFLRNYTDLVASVYAADDTERTNPLWESQPQSGNKNFYSGDPKNPKSSIIYPTEWN 959
PrtS_DGCC8014      VFKGVFLRNYTDLVASVYAADDTERTNPLWESQPQSGNKNFYSGDPKNPKSSIIYPTEWN 959
PrtS_DGCC7854      VFKGVFLRNYTDLVASVYAADDTERTNPLWESQPQSGNKNFYSGDPKNPKSSIIYPTEWN 927
                   .************************************:**:***:***************

PrtS_DGCC715       GTDSEGNALADGKYQYVLTYSSEVPGAAVQTMIFDVIIDRESPVITTATYDETNFTFNPR 988
PrtS_LMD-9         GTDSEGNALADGKYQYVLTYSSEVPGAAVQTMIFDVIIDRESPVITTATYDETNFTFNPR 1020
PrtS_DGCC855       GTDSEGNALADGKYQYVLTYSSEVPGAAVQTMIFDVIIDRESPVITTATYDETNFTFNPR 1020
PrtS_DGCC7853      GTDSEGNALADGKYQYVLTYSSEVPGAAVQTMIFDVIIDRESPVITTATYDETNFTFNPR 1020
PrtS_DGCC7984      GTDSEGNALADGKYQYVLTYSSEVPGAAVQTMIFDVIIDRESPVITTATYDETNFTFNPR 1020
PrtS_JIM8232       GTDSEGNALADGKYQYVLTYSSEVPGAAVQTMIFDVIIDRESPVITTATYDETNFTFNPR 1020
PrtS_DGCC7710      GTDSEGNALADGKYQYVLTYSSEVPGAAVQTMIFDVIIDRESPVITTATYDETNFTFNPR 1020
PrtS_DGCC7879      GTDSEGNALADGKYQYVLTYSSEVPGAAVQTMIFDVIIDRESPVITTATYDETNFTFNPR 1020
PrtS_CNRZ385       GTDSDGNALADGKYQYVLTYSSKVPGAAVQTMIFDVIIDRESPVITTATYDETNFTFNPR 987
PrtS_DGCC2057      GTDSDGNALADGKYQYVLTYSSKVPGAAVQTMIFDVIIDRESPVITTATYDETNFTFNPR 1019
PrtS_DGCC7809      GTDSDGNALADGKYQYVLTYSSKVPGAAVQTMIFDVIIDRESPVITTATYDETNFTFNPR 1019
PrtS_DGCC7796      GTDSEGNALADGKYQYVLTYSSEVPGAAVQTMIFDVIIDRESPVITTATYDETNFTFNPR 1019
PrtS_DGCC8014      GTDSEGNALADGKYQYVLTYSSEVPGAAVQTMIFDVIIDRESPVITTATYDETNFTFNPR 1019
PrtS_DGCC7854      GTDSEGNALADGKYQYVLTYSSEVPGAAVQTMIFDVIIDRESPVITTATYDETNFTFNPR 987
                   ****:*****************:*************************************
```

```
PrtS_DGCC715       PAIEKGESGLYREQVFYLVADASGVTTIPSLLENGDVTVSDNKVFVAQNDDGSFTLPLDL 1048
PrtS_LMD-9         PAIEKGESGLYREQVFYLVADASGVTTIPSLLENGDVTVSDNKVFVAQNDDGSFTLPLDL 1080
PrtS_DGCC855       PAIEKGESGLYREQVFYLVADASGVTTIPSLLENGDVTVSDNKVFVAQNDDGSFTLPLDL 1080
PrtS_DGCC7853      PAIEKGESGLYREQVFYLVADASGVTTIPSLLENGDVTVSDNKVFVAQNDDGSFTLPLDL 1080
PrtS_DGCC7984      PAIEKGESGLYREQVFYLVADASGVTTIPSLLENGDVTVSDNKVFVAQNDDGSFTLPLDL 1080
PrtS_JIM8232       PAIEKGESGLYREQVFYLVADASGVTTIPSLLENGDVTVSDNKVFVAQNDDGSFTLPLDL 1080
PrtS_DGCC7710      PAIEKGESGLYREQVFYLVADASGVTTIPSLLENGDVTVSDNKVFVAQNDDGSFTLPLDL 1080
PrtS_DGCC7879      PAIEKGESGLYREQVFYLVADASGVTTIPSLLENGDVTVSDNKVFVAQNDDGSFTLPLDL 1080
PrtS_CNRZ385       PAIEKGESGLYREQVFYLVADASGVTTIPSLLKNGDVTVSDNKVFVAQNDDGSFTLPLDL 1047
PrtS_DGCC2057      PAIEKGESGLYREQVFYLVADASGVTTIPSLLKNGDVTVSDNKVFVAQNDDGSFTLPLDL 1079
PrtS_DGCC7809      PAIEKGESGLYREQVFYLVADASGVTTIPSLLKNGDVTVSDNKVFVAQNDDGSFTLPLDL 1079
PrtS_DGCC7796      PAIEKGESGLYREQVFYLVADASGVTTIPSLLKNGDVTVSDNKVFVAQNDDGSFTLPLDL 1079
PrtS_DGCC8014      PAIEKGESGLYREQVFYLVADASGVTTIPSLLKNGDVTVSDNKVFVAQNDDGSFTLPLDL 1079
PrtS_DGCC7854      PAIEKGESGLYREQVFYLVADASGVTTIPSLLKNGDVTVSDNKVFVAQNDDGSFTLPLDL 1047
                   ********************************:***************************

PrtS_DGCC715       ADISKFYYTVEDYAGNISYEKVENLISIGNEKGLVTVNILDKDTNSPVPILFSYSVTDET 1108
PrtS_LMD-9         ADISKFYYTVEDYAGNISYEKVENLISIGNEKGLVTVNILDKDTNSPVPILFSYSVTDET 1140
PrtS_DGCC855       ADISKFYYTVEDYAGNISYEKVENLISIGNEKGLVTVNILDKDTNSPVPILFSYSVTDET 1140
PrtS_DGCC7853      ADISKFYYTVEDYAGNISYEKVENLISIGNEKGLVTVNILDKDTNSPVPILFSYSVTDET 1140
PrtS_DGCC7984      ADISKFYYTVEDYAGNISYEKVENLISIGNEKGLVTVNILDKDTNSPVPILFSYSVTDEI 1140
PrtS_JIM8232       ADISKFYYTVEDYAGNISYEKVENLISIGNEKGLVTVNILDKDTNSPVPILFSYSVTDEI 1140
PrtS_DGCC7710      ADISKFYYTVEDYAGNISYEKVENLISIGNEKGLVTVNILDKDTNSPVPILFSYSVTDEI 1140
PrtS_DGCC7879      ADISKFYYTVEDYAGNISYEKVENLISIGNEKGLVTVNILDKDTNSPVPILFSYSVTDEI 1140
PrtS_CNRZ385       ADISKFYYTVEDYAGNISYEKVENLISIGNEKGLVTVNILDKDTNSPVPILFSYSVTDET 1107
PrtS_DGCC2057      ADISKFYYTVEDYAGNISYEKVENLISIGNEKGLVTVNILDKDTNSPVPILFSYSVTDET 1139
PrtS_DGCC7809      ADISKFYYTVEDYAGNISYEKVENLISIGNEKGLVTVNILDKDTNSPVPILFSYSVTDET 1139
PrtS_DGCC7796      ADISKFYYTVEDYAGNISYEKVENLISIGNEKGLVTVNILDKDTNSPVPILFSYSVTDET 1139
PrtS_DGCC8014      ADISKFYYTVEDYAGNISYEKVENLISIGNEKGLVTVNILDKDTNSPVPILFSYSVTDET 1139
PrtS_DGCC7854      ADISKFYYTVEDYAGNISYEKVENLISIGNEKGLVTVNILDKDTNSPVPILFSYSVTDET 1107
                   *********************************************************** 

PrtS_DGCC715       GKIVAELPRYAGDTSVLKLPFGTYTFDLFLYDTEWSSLAGETKAVVTILEDNSTAEVNFY 1168
PrtS_LMD-9         GKIVAELPRYAGDTSVLKLPFGTYTFDLFLYDTEWSSLAGETKAVVTILEDNSTAEVNFY 1200
PrtS_DGCC855       GKIVAELPRYAGDTSVLKLPFGTYTFDLFLYDTEWSSLAGETKAVVTILEDNSTAEVNFY 1200
PrtS_DGCC7853      GKIVAELPRYAGDTSVLKLPFGTYTFDLFLYDTEWSSLAGETKAVVTILEDNSTAEVNFY 1200
PrtS_DGCC7984      GKIVAELPRYAGDTSVLKLPFGTYTFDLFLYDTEWSSLAGETKAVVTISEENSTAEVNFY 1200
PrtS_JIM8232       GKIVAELPRYAGDTSVLKLPFGTYTFDLFLYDTEWSSLAGETKAVVTISEENSTAEVNFY 1200
PrtS_DGCC7710      GKIVAELPRYAGDTSVLKLPFGTYTFDLFLYDTEWSSLAGETKAVVTISEENSTAEVNFY 1200
PrtS_DGCC7879      GKIVAELPRYAGDTSVLKLPFGTYTFDLFLYDTEWSSLAGETKAVVTISEENSTAEVNFY 1200
PrtS_CNRZ385       GKIVAELPRYAGDTSVLKLPFGTYTFDLFLYDTEWSSLAGETKAVVTILEDNSTAEVNFY 1167
PrtS_DGCC2057      GKIVAELPRYAGDTSVLKLPFGTYTFDLFLYDTEWSSLAGETKAVVTILEDNSTAEVNFY 1199
PrtS_DGCC7809      GKIVAELPRYAGDTSVLKLPFGTYTFDLFLYDTEWSSLAGETKAVVTILEDNSTAEVNFY 1199
PrtS_DGCC7796      GKIVAELPRYAGDTSVLKLPFGTYTFDLFLYDTERSSLAGETKAVVTILEDNSTAEVNFY 1199
PrtS_DGCC8014      GKIVAELPRYAGDTSVLKLPFGTYTFDLFLYDTERSSLAGETKAVVTILEDNSTAEVNFY 1199
PrtS_DGCC7854      GKIVAELPRYAGDTSVLKLPFGTYTFDLFLYDTERSSLAGETKAVVTILEDNSTAEVNFY 1167
                   ********************************** ************* *:*********

PrtS_DGCC715       VTLKDKANLLIDIDALLPSGSTIQLVTADGQAIQLPNAKYSKTDYGKFVPVGTYTILPTL 1228
PrtS_LMD-9         VTLKDKANLLIDIDALLPSGSTIQLVTADGQAIQLPNAKYSKTDYGKFVPVGTYTILPTL 1260
PrtS_DGCC855       VTLKDKANLLIDIDALLPSGSTIQLVTADGQAIQLPNAKYSKTDYGKFVPVGTYTILPTL 1260
PrtS_DGCC7853      VTLKDKANLLIDIDALLPSGSTIQLVTADGQAIQLPNAKYSKTDYGKFVPVGTYTILPTL 1260
PrtS_DGCC7984      VTLKDKANLLVDIDALLPSGSTIQLVTADGQTIQLPNAKYSKTDYGKFVPVGTYTILPTL 1260
PrtS_JIM8232       VTLKDKANLLVDIDALLPSGSTIQLVTADGQTIQLPNAKYSKTDYGKFVPVGTYTILPTL 1260
PrtS_DGCC7710      VTLKDKANLLVDIDALLPSGSTIQLVTADGQTIQLPNAKYSKTDYGKFVPVGTYTILPTL 1260
PrtS_DGCC7879      VTLKDKANLLVDIDALLPSGSTIQLVTADGQTIQLPNAKYSKTDYGKFVPVGTYTILPTL 1260
PrtS_CNRZ385       VTLKDKANLLIDIDALLPSGSTIQLVTADGQAIQLPNAKYSKTDYGKFVPVGTYTILPTL 1227
PrtS_DGCC2057      VTLKDKANLLIDIDALLPSGSTIQLVTADGQAIQLPNAKYSKTDYGKFVPVGTYTILPTL 1259
PrtS_DGCC7809      VTLKDKANLLIDIDALLPSGSTIQLVTADGQAIQLPNAKYSKTDYGKFVPVGTYTILPTL 1259
PrtS_DGCC7796      VTLKDKANLLIDIDALLPSGSTIQLVTADGQAIQLPNAKYSKTDYGKFVPVGTYTILPTL 1259
PrtS_DGCC8014      VTLKDKANLLIDIDALLPSGSTIQLVTADGQAIQLPNAKYSKTDYGKFVPVGTYTILPTL 1259
PrtS_DGCC7854      VTLKDKANLLIDIDALLPSGSTIQLVTADGQAIQLPNAKYSKTDYGKFVPVGTYTILPTL 1227
                   **********:********************:****************************

PrtS_DGCC715       PEGYEFLEELDVAVLANQSNVKKLTLINKVALKELIAELAGLEETARYYNASPELQTAYA 1288
PrtS_LMD-9         PEGYEFLEELDVAVLANQSNVKKLTLINKVALKELIAELAGLEETARYYNASPELQTAYA 1320
PrtS_DGCC855       PEGYEFLEELDVAVLANQSNVKKLTLINKVALKELIAELAGLEETARYYNASPELQTAYA 1320
PrtS_DGCC7853      PEGYEFLEELDVAVLANQSNVKKLTLINKVALKELIAELAGLEETARYYNASPELQTAYA 1320
```

```
PrtS_DGCC7984      PEGYEFLEELDVAVLANQSNVKKLTLINKVALKKLIAELAGLEETARYYNASPELQTAYA 1320
PrtS_JIM8232       PEGYEFLEELDVAVLANQSNVKKLTLINKVALKKLIAELAGLEETARYYNASPELQTAYA 1320
PrtS_DGCC7710      PEGYEFLEELDVAVLANQSNVKKLTLINKVALKKLIAELAGLEETARYYNASPELQTAYA 1320
PrtS_DGCC7879      PEGYEFLEELDVAVLANQSNVKKLTLINKVALKKLIAELAGLEETARYYNASPELQTAYA 1320
PrtS_CNRZ385       PEGYEFLEELDVAVLANQSNVKKLTLINKVALKELIAELAGLEETARYYNASPELQTAYA 1287
PrtS_DGCC2057      PEGYEFLEELDVAVLANQSNVKKLTLINKVALKELIAELAGLEETARYYNASPELQTAYA 1319
PrtS_DGCC7809      PEGYEFLEELDVAVLANQSNVKKLTLINKVALKELIAELAGLEETARYYNASPELQTAYA 1319
PrtS_DGCC7796      PEGYEFLEELDVAVLANQSNVKKLTLINKVALKELIAELAGLEETARYYNASPELQTAYA 1319
PrtS_DGCC8014      PEGYEFLEELDVAVLANQSNVKKLTLINKVALKELIAELAGLEETARYYNASPELQTAYA 1319
PrtS_DGCC7854      PEGYEFLEELDVAVLANQSNVKKLTLINKVALKELIAELAGLEETARYYNASPELQTAYA 1287
                   *********************************:**************************

PrtS_DGCC715       KALEDANAVYANKHNQAQVDSALASLVAAREQLNGQATDKEKLIAEVSNYTPTQANFIYY 1348
PrtS_LMD-9         KALEDANAVYANKHNQAQVDSALASLVAAREQLNGQATDKEKLIAEVSNYTPTQANFIYY 1380
PrtS_DGCC855       KALEDANAVYANKHNQAQVDSALASLVAAREQLNGQATDKEKLIAEVSNYTPTQANFIYY 1380
PrtS_DGCC7853      KALEDANAVYANKHNQAQVDSALASLVAAREQLNGQATDKEKLIAEVSNYTPTQANFIYY 1380
PrtS_DGCC7984      KALEDANAVYANKHNQAQVDSTLASLVAAREQLNGQATDKEKLIAEVSNYTPTQANFIYF 1380
PrtS_JIM8232       KALEDANAVYANKHNQAQVDSTLASLVAAREQLNGQATDKEKLIAEVSNYTPTQANFIYF 1380
PrtS_DGCC7710      KALEDANAVYANKHNQAQVDSTLASLVAAREQLNGQATDKEKLIAEVSNYTPTQANFIYF 1380
PrtS_DGCC7879      KALEDANAVYANKHNQAQVDSTLASLVAAREQLNGQATDKEKLIAEVSNYTPTQANFIYF 1380
PrtS_CNRZ385       KALEDANAVYANKHNQAQVDSALASLVAAREQLNGQATDKEKLIAEVSNYTPTQANFIYY 1347
PrtS_DGCC2057      KALEDANAVYANKHNQAQVDSALASLVAAREQLNGQATDKEKLIAEVSNYTPTQANFIYY 1379
PrtS_DGCC7809      KALEDANAVYANKHNQAQVDSALASLVAAREQLNGQATDKEKLIAEVSNYTPTQANFIYY 1379
PrtS_DGCC7796      KALEDANAVYANKHNQAQVDSALASLVAAREQLNGQATDKEKLIAEVSNYTPTQANFIYY 1379
PrtS_DGCC8014      KALEDANAVYANKHNQAQVDSALASLVAAREQLNGQATDKEKLIAEVSNYTPTQANFIYY 1379
PrtS_DGCC7854      KALEDANAVYANKHNQAQVDSALASLVAAREQLNGQATDKEKLIAEVSNYTPTQANFIYY 1347
                   *********************:*************************************:

PrtS_DGCC715       NAENTKQIAYDTAVRSAQLVLNQENVTQAVVNQALADLLAAKANLDGQKTDISALRSAVS 1408
PrtS_LMD-9         NAENTKQIAYDTAVRSAQLVLNQENVTQAVVNQALADLLAAKANLDGQKTDISALRSAVS 1440
PrtS_DGCC855       NAENTKQIAYDTAVRSAQLVLNQENVTQAVVNQALADLLAAKANLDGQKTDISALRSAVS 1440
PrtS_DGCC7853      NAENTKQIAYDTAVRSAQLVLNQENVTQAVVNQALADLLAAKANLDGQKTDISALRSAVS 1440
PrtS_DGCC7984      NAENTKQIAYDTAVRSAQLVLNQENVTQAVVNQALADLLAAKAGLDGQKTDISALRSAVS 1440
PrtS_JIM8232       NAENTKQIAYDTAVRSAQLVLNQENVTQAVVNQALADLLAAKAGLDGQKTDISALRSAVS 1440
PrtS_DGCC7710      NAENTKQIAYDTAVRSAQLVLNQENVTQAVVNQALADLLAAKAGLDGQKTDISALRSAVS 1440
PrtS_DGCC7879      NAENTKQIAYDTAVRSAQLVLNQENVTQAVVNQALADLLAAKAGLDGQKTDISALRSAVS 1440
PrtS_CNRZ385       NAENTKQIAYDTAVRSAQLVLNQENVTQAVVNQALADLLAAKANLDGQKTDISALRSAVS 1407
PrtS_DGCC2057      NAENTKQIAYDTAVRSAQLVLNQENVTQAVVNQALADLLAAKANLDGQKTDISALRSAVS 1439
PrtS_DGCC7809      NAENTKQIAYDTAVRSAQLVLNQENVTQAVVNQALADLLAAKANLDGQKTDISALRSAVS 1439
PrtS_DGCC7796      NAENTKQIAYDTAVRSAQLVLNQENVTQAVVNQALADLLAAKANLDGQKTDISALRSAVS 1439
PrtS_DGCC8014      NAENTKQIAYDTAVRSAQLVLNQENVTQAVVNQALADLLAAKANLDGQKTDISALRSAVS 1439
PrtS_DGCC7854      NAENTKQIAYDTAVRSAQLVLNQENVTQAVVNQALADLLAAKANLDGQKTDISALRSAVS 1407
                   *******************************************.****************

PrtS_DGCC715       VSSVLKATDAKYLNASENVKQAYDQAVEAAKAILVDESASQASVDQALAVLTSAQAELDG 1468
PrtS_LMD-9         VSSVLKATDAKYLNASENVKQAYDQAVEAAKAILVDESASQASVDQALAVLTSAQAELDG 1500
PrtS_DGCC855       VSSVLKATDAKYLNASENVKQAYDQAVEAAKAILVDESASQASVDQALAVLTSAQAELDG 1500
PrtS_DGCC7853      VSSVLKATDAKYLNASENVKQAYDQAVEAAKAILVDESASQASVDQALAVLTSAQAELDG 1500
PrtS_DGCC7984      VSSVLKATDAKYLNASENVKQAYDQAVEAAKAILVDESASQASVDQALAVLTSAQAELDG 1500
PrtS_JIM8232       VSSVLKATDAKYLNASENVKQAYDQAVEAAKAILADESASQASVDQALAVLTSAQAELDG 1500
PrtS_DGCC7710      VSSVLKATDAKYLNASENVKQAYDQAVEAAKAILVDESASQASVDQALAVLTSAQAELDG 1500
PrtS_DGCC7879      VSSVLKATDAKYLNASENVKQAYDQAVEAAKAILVDESASQASVDQALAVLTSAQAELDG 1500
PrtS_CNRZ385       VSSVLKATDAKYLNASENVKQAYDQAVEAAKAILVDESASQASVDQALAVLTSAQAELDG 1467
PrtS_DGCC2057      VSSVLKATDAKYLNASENVKQAYDQAVEAAKAILVDESASQASVDQALAVLTSAQAELDG 1499
PrtS_DGCC7809      VSSVLKATDAKYLNASENVKQAYDQAVEAAKAILVDESASQASVDQALAVLTSAQAELDG 1499
PrtS_DGCC7796      VSSVLKATDAKYLNASENVKQAYDQAVEAAKAILVDESASQASVDQALAVLTSAQAELDG 1499
PrtS_DGCC8014      VSSVLKATDAKYLNASENVKQAYDQAVEAAKAILVDESASQASVDQALAVLTSAQAELDG 1499
PrtS_DGCC7854      VSSVLKATDAKYLNASENVKQAYDQAVEAAKAILVDESASQASVDQALAVLTSAQAELDG 1467
                   **********************************.*************************

PrtS_DGCC715       VATSTNDAKEPANTATDKKDEGTVTPPPIDSEIVDVQAPPVKDTGNSEHVPIGQKPNPQP 1528
PrtS_LMD-9         VATSTNDAKEPANTATDKKDEGTVTPPPIDSEIVDVQAPPVKDTGNSEHVPIGQKPNPQP 1560
PrtS_DGCC855       VATSTNDAKEPANTATDKKDEGTVTPPPIDSEIVDVQAPPVKDTGNSEHVPIGQKPNPQP 1560
PrtS_DGCC7853      VATSTNDAKEPANTATDKKDEGTVTPPPIDSEIVDVQAPPVKDTGNSEHVPIGQKPNPQP 1560
PrtS_DGCC7984      VATSTNDAKEPANTATDKKDEGTVTPPPIDSEIVDVQAPPVKDTGNSEHVPIGQKPNPQP 1560
PrtS_JIM8232       VATSTNDAKEPANTATDKKDEGTVTPPPIDSEKVDVQAPPVKDTGNSGHVPIGQKPNPQP 1560
PrtS_DGCC7710      VATSTNDAKEPANTATDKKDEGTVTPPPIDSEIVDVQAPPVKDTGNSEHVPIGQKPNPQP 1560
PrtS_DGCC7879      VATSTNDAKEPANTATDKKDEGTVTPPPIDSEIVDVQAPPVKDTGNSEHVPIGQKPNPQP 1560
```

```
PrtS_CNRZ385       VATSTNDAKEPANTATDKKDEGTVTPPPIDSEIVDVQAPPVKDTGNSEHVPIGQKPNPQP 1527
PrtS_DGCC2057      VATSTNDAKEPANTATDKKDEGTVTPPPIDSEIVDVQAPPVKDTGNSEHVPIGQKPNPQP 1559
PrtS_DGCC7809      VATSTNDAKEPANTATDKKDEGTVTPPPIDSEIVDVQAPPVKDTGNSEHVPIGQKPNPQP 1559
PrtS_DGCC7796      VATSTNDAKEPANTATDKKDEGTVTPPPIDSEIVDVQAPPVKDTGNSEHVPIGQKPNPQP 1559
PrtS_DGCC8014      VATSTNDAKEPANTATDKKDEGTVTPPPIDSEIVDVQAPPVKDTGNSEHVPIGQKPNPQP 1559
PrtS_DGCC7854      VATSTNDAKEPANTATDKKDEGTVTPPPIDSEIVDVQAPPVKDTGNSEHVPISQKPNPQP 1527
                   ******************************** ************** ****.*******

PrtS_DGCC715       TLPRPVTLQASLSSPNQEKQVTQLPNTGENDTKYYLVPGVIIGLGTLLVSIRRHKEEV 1586
PrtS_LMD-9         TLPRPVTLQASLSSPNQEKQVTQLPNTGENDTKYYLVPGVIIGLGTLLVSIRRHKEEV 1618
PrtS_DGCC855       TLPRPVTLQASLSSPNQEKQVTQLPNTGENDTKYYLVPGVIIGLGTLLVSIRRHKEEV 1618
PrtS_DGCC7853      TLPRPVTLQASLSSPNQEKQVTQLPNTGENDTKYYLVPGVIIGLGTLLVSIRRHKEEV 1618
PrtS_DGCC7984      TLPRPVTLQASLSSPNQEKQVTQLPNTGENDTKYYLVPGVIIGLGTLLVSIRRHKEEV 1618
PrtS_JIM8232       TLPRPVTLQASLSSPNQEKQVTQLPNTGENDTRYYLVLGVIIGLGTLLVSKRRHKEEV 1618
PrtS_DGCC7710      TLPRPVTLQASLSSPNQEKQVTQLPNTGENDTKYYLVPGVIIGLGTLLVSIRRHKEEV 1618
PrtS_DGCC7879      TLPRPVTLQASLSSPNQEKQVTQLPNTGENDTKYYLVPGVIIGLGTLLVSIRRHKEEV 1618
PrtS_CNRZ385       TLPRPVTLQASLSSPNQEKQVTQLPNTGENDTKYYLVPGVIIGLGTLLVSIRRHKEEV 1585
PrtS_DGCC2057      TLPRPVTLQASLSSPNQEKQVTQLPNTGENDTKYYLVPGVIIGLGTLLVSIRRHKEEV 1617
PrtS_DGCC7809      TLPRPVTLQASLSSPNQEKQVTQLPNTGENDTKYYLVPGVIIGLGTLLVSIRRHKEEV 1617
PrtS_DGCC7796      TLPRPVTLQASLSSPNQEKQVTQLPNTGENDTKYYLVPGVIIGLGTLLVSIRRHKEEV 1617
PrtS_DGCC8014      TLPRPVTLQASLSSPNQEKQVTQLPNTGENDTKYYLVPGVIIGLGTLLVSIRRHKEEV 1617
PrtS_DGCC7854      TLPRPVTLQASLSSPNQEKQVTQLPNTGENDTKYYLVPGVIIGLGTLLVSIRRHKEEV 1585
                   ********************************:**** ************ *******
```

Figure S2. Multiple sequence alignment of PrtS from *S. thermophilus*. Sequence alignment was performed with ClustalW2 package with default parameters except gap open and gap extension parameters fixed at 100 and 10, respectively (http://www.ebi.ac.uk/Tools/msa/clustalw2/). The catalytic triad described by Bonifait et al and the LPXTG motif are highlighted in yellow and red, respectively [1]. The signal peptide and the propeptide, previously identified by Fernandez-Espla are indicated by green and blue lines above the PrtS sequence, respectively [2]. Genebank accession numbers: *S. thermophilus* LMD-9, ABJ66087; CNRZ385, AF243528; JIM8232, FJ200299.

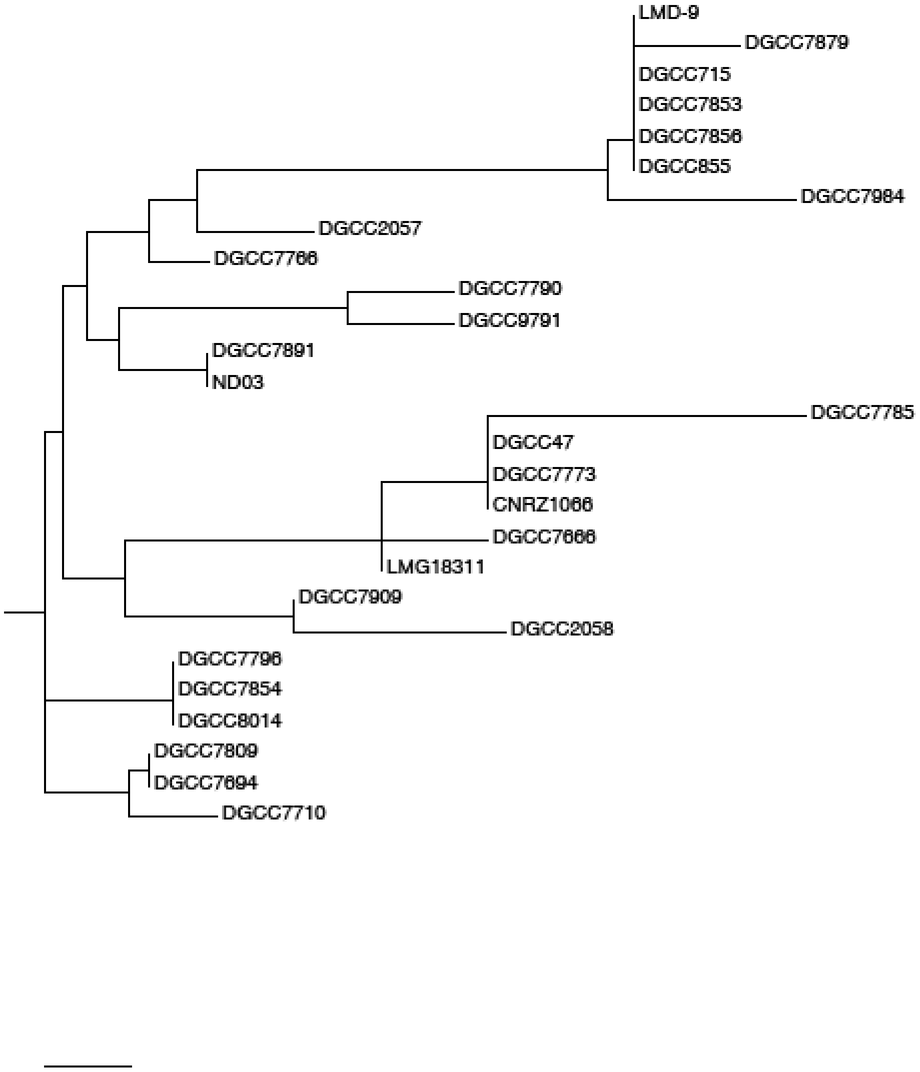

Figure S3. Phylogenetic tree constructed from the amino acid sequences of SrtA proteins from *S. thermophilus*. Sequence alignment and phylogenetic tree (neighbour-joining method) were performed using the ClustalW2 package with default parameters (http://www.ebi.ac.uk/Tools/msa/clustalw2/). Genebank accession numbers: *S. thermophilus* LMD-9, YP_820636.1; ND03, ADQ63270. For the alignment purposes, we have considered the SrtA sequences upstream and downstream of the nonsense mutation of LMG18311, CNRZ1066, DGCC7785, DGCC47, DGCC7773, DGCC7666 and DGCC7909.

```
LMD-9           MRKDNKNKTPKKHRKWLEVLRWILIVVLLVVGLALIFNKSIRNTIIAWNTNKYQVSKVSK 60
DGCC7853        MRKDNKNKTPKKHRKWLEVLRWILIVVLLVVGLALIFNKSIRNTIIAWNTNKYQVSKVSK 60
DGCC7856        MRKDNKNKTPKKHRKWLEVLRWILIVVLLVVGLALIFNKSIRNTIIAWNTNKYQVSKVSK 60
DGCC855         MRKDNKNKTPKKHRKWLEVLRWILIVVLLVVGLALIFNKSIRNTIIAWNTNKYQVSKVSK 60
DGCC7879        MRKDNKNKTPKKHRKWLEVLRWILIVVLLVVGLALIFNKSIRNTIIAWNTNKYQVSKVSK 60
DGCC7984        MRKYNKNKTPKKHRRWLEVLRWILIVVLLVVGLALIFNKSIRNTIIAWNTNKYQVSKVSK 60
DGCC2057        MRKDNKNKTPKKHRKWLEVLRWILIVVLLVVGLALIFNKSIRNTIIAWNTNKYQVSKVSK 60
DGCC7766        MRKDNKNKTPKKHRKWLEVLRWILIVVLLVVGLALIFNKSIRNTIIAWNTNKYQVSKVSK 60
DGCC7790        MRKDNKNKTPKKHRKWLEVLRWILIVVLLVVGLALIFNKSIRNTIIAWNTNKYQVSKVSK 60
DGCC9791        MRKDNKNKTPKKHCKWLEVLRWILIVVLLVVGLALIFNKSIRNTIIAWNTNKYQVSKVSK 60
DGCC7891        MRKYNKNKTPKKHRKWLEVLRWILIVVLLVVGLALIFNKSIRNTIIAWNTNKYQVSKVSK 60
ND03            MRKYNKNKTPKKHRKWLEVLRWILIVVLLVVGLALIFNKSIRNTIIAWNTNKYQVSKVSK 60
DGCC7809        MRKDNKNKTPKKHRKWLEVLRWILIVVLLVVGLALILNKSIRNTIIAWNTNKYQVSKVSK 60
DGCC7694        MRKDNKNKTPKKHRKWLEVLRWILIVVLLVVGLALILNKSIRNTIIAWNTNKYQVSKVSK 60
DGCC7710        MRKYNKNKTPKKHRKWLEVLRWILIVVLLVVGLALILNKSIRNTIIAWNTNKYQVSKVSK 60
DGCC7796        MRKDNKNNTPKKHRKWLEVLRWILIVVLLVVGLALIFNKSIRNTIIAWNTNKYQVSKVSK 60
DGCC7854        MRKDNKNNTPKKHRKWLEVLRWILIVVLLVVGLALIFNKSIRNTIIAWNTNKYQVSKVSK 60
DGCC8014        MRKDNKNNTPKKHRKWLEVLRWILIVVLLVVGLALIFNKSIRNTIIAWNTNKYQVSKVSK 60
DGCC7909        MRKDNKNKTPKKHRKWLEVLRWILIVVLLVVGLALIFNKSIRNTIIAWNTNKYQVSKVSK 60
DGCC2058        MRKDNKNKTPKKHRKWLEVLRWFLIVVLLVVGLALIFNKSIRNTIIAWNTNKYQVSKVSE 60
DGCC7785        MRKDNKNKTPKKHRKWLEVLRWILIVVLLVVGLALISNKLIRNTIIAWNTNKYQVSKVSK 60
DGCC47          MRKDNKNKTPKKHRKWLEVLRWILIVVLLVVGLALISNKLIRNTIIAWNTNKYQVSKVSK 60
DGCC7773        MRKDNKNKTPKKHRKWLEVLRWILIVVLLVVGLALISNKLIRNTIIAWNTNKYQVSKVSK 60
CNRZ1066        MRKDNKNKTPKKHRKWLEVLRWILIVVLLVVGLALISNKLIRNTIIAWNTNKYQVSKVSK 60
LMG18311        MRKDNKNKTPKKHRKWLEVLRWILIVVLLVVGLALISNKLIRNTIIAWNTNKYQVSKVSK 60
DGCC7666        MRKDNKNKTPKKHRKWLEVLRWILIVVLLVVGLALISNKLIRNTIIAWNTNKYQVSKVSK 60
                *** ***:***** :*******:************* ** *******************:

LMD-9           KTIKKNKEAKASYDFDTVKSVSTESVLQAQMGSQKLPVVGGIAIPEVGINLPIFKGLGNT 120
DGCC7853        KTIKKNKEAKASYDFDTVKSVSTESVLQAQMGSQKLPVVGGIAIPEVGINLPIFKGLGNT 120
DGCC7856        KTIKKNKEAKASYDFDTVKSVSTESVLQAQMGSQKLPVVGGIAIPEVGINLPIFKGLGNT 120
DGCC855         KTIKKNKEAKASYDFDTVKSVSTESVLQAQMGSQKLPVVGGIAIPEVGINLPIFKGLGNT 120
DGCC7879        KTIKKNKEAKASYDFDTVKSVSTESVLQAQMGSQKLPVVGGIAIPEVGINLPIFKGLGNT 120
DGCC7984        KTIKKNKEAKASYDFDTVKSVSTESVLQAQMGSQKLPVVGGIAIPEVGINLPIFKGLGNT 120
DGCC2057        KTIKKNKEAKASYDFDTVKSVSTESVLQAKMGSQKLPVVGGIAIPEVGINLPIFKGLGNT 120
DGCC7766        KTIKKNKEAKASYDFDTVKSVSTESVLQAQMGSQKLPVVGGIAIPEVGINLPIFKGLGNT 120
DGCC7790        KTIKKNKEARASYDFDTVKSVSTESVLQAQMGSQKLPVVGGIAIPEVGINLPIFKGLGNT 120
DGCC9791        KTIKKNKEARASYDFDTVKSVSTESVLQAQMGSQKLPVVGGIAIPEVGINLPIFKGLGNT 120
DGCC7891        KTIKKNKEARASYDFDTVKSVSTESVLQAQMGSQKLPVVGGIAIPEVGINLPIFKGLGNT 120
ND03            KTIKKNKEARASYDFDTVKSVSTESVLQAQMGSQKLPVVGGIAIPEVGINLPIFKGLGNT 120
DGCC7809        KTIKKNKEARASYDFDTVKSVSTESVLQAQMGSQKLPVVGGIAIPEVGINLPIFKGLGNT 120
DGCC7694        KTIKKNKEARASYDFDTVKSVSTESVLQAQMGSQKLPVVGGIAIPEVGINLPIFKGLGNT 120
DGCC7710        KTIKKNKEARASYDFDTVKSVSTESVLQAQMGSQKLPVVGGIAIPEVGINLPIFKGLGNT 120
DGCC7796        KTIKKNKEARASYDFDTVKSVSTESVLQAQMGSQKLPVVGGIAIPEVGINLPIFKGLGNT 120
DGCC7854        KTIKKNKEARASYDFDTVKSVSTESVLQAQMGSQKLPVVGGIAIPEVGINLPIFKGLGNT 120
DGCC8014        KTIKKNKEARASYDFDTVKSVSTESVLQAQMGSQKLPVVGGIAIPEVGINLPIFKGLGNT 120
DGCC7909        KTIKKNKKARASYDFYTVKSVSTESVLQAQMGSQKLPVVGGIAIPEVGINLPIFKGLGNT 120
DGCC2058        KTIKKNKKARASYDFYTVKSVSTESVLQAQMGSQKLPVVGGIAIPEVGINLPIFKGLGNT 120
DGCC7785        KTIKKNKEARASYDFYTVKSVSTESVLQAQMGSQKLPVVGGIAIPEVGINLPNFKGLXNT 120
DGCC47          KTIKKNKEARASYDFYTVKSVSTESVLQAQMGSQKLPVVGGIAIPEVGINLPIFKGLGNT 120
DGCC7773        KTIKKNKEARASYDFYTVKSVSTESVLQAQMGSQKLPVVGGIAIPEVGINLPIFKGLGNT 120
CNRZ1066        KTIKKNKEARASYDFYTVKSVSTESVLQAQMGSQKLPVVGGIAIPEVGINLPIFKGLGNT 120
LMG18311        KTIKKNKEARASYDFYTVKSVSTESVLQAQMGSQKLPVVGGIAIPEVGINLPIFKGLGNT 120
DGCC7666        KTIKKNKEARASYDFYTVKSVSTGSVLQAQMGSQKLPVVGGIAIPEVGINLPIFKGLGNT 120
                *******:*:***** ******* *****:********************** **** **

LMD-9           ELTYGAGTMKENQVMGGENNYSLASHHIFGIAGASDMLFSPLDRAKNGMKIYLTDKNKIY 180
DGCC7853        ELTYGAGTMKENQVMGGENNYSLASHHIFGIAGASDMLFSPLDRAKNGMKIYLTDKNKIY 180
DGCC7856        ELTYGAGTMKENQVMGGENNYSLASHHIFGIAGASDMLFSPLDRAKNGMKIYLTDKNKIY 180
DGCC855         ELTYGAGTMKENQVMGGENNYSLASHHIFGIAGASDMLFSPLDRAKNGMKIYLTDKNKIY 180
DGCC7879        ELTYGAGTMKENQVMGGENNYSLASHHIFGIAGASDMLFSPLDRAKNGMKIYLTDKNKIY 180
DGCC7984        ELTYGAGTMKENQVMGGENNYSLASHHIFGIAGASDMLFSPLDRAKNGMKIYLTDKNKIY 180
DGCC2057        ELTYGAGTMKENQVMGGENNYSLASHHIFGIAGASDMLFSPIDRAKNGMKIYLTDKNKVY 180
DGCC7766        ELTYGAGTMKENQVMGGENNYSLASHHIFGIAGASDMLFSPIDRAKNGMKIYLTDKNKVY 180
DGCC7790        ELTYGAGTMKENQVMGGENNYSLASHHIFGIAGASDMLFSPIDRAKNGMKIYLTDKNKVY 180
DGCC9791        ELTYGAGTMKENQVMGGENNYSLASHHIFGIAGASDMLFSPIDRAKNGMKIYLTDKNKVY 180
DGCC7891        ELTYGAGTMKENQVMGGENNYSLASHHIFGIAGASDMLFSPIDRAKNGMKIYLTDKNKVY 180
ND03            ELTYGAGTMKENQVMGGENNYSLASHHIFGIAGASDMLFSPIDRAKNGMKIYLTDKNKVY 180
```

```
DGCC7809        ELTYGAGTMKENQVMGGENNYSLASHHIFGIAGASDMLFSPIDRAKNGMKIYLTDKNKVY 180
DGCC7694        ELTYGAGTMKENQVMGGENNYSLASHHIFGIAGASDMLFSPIDRAKNGMKIYLTDKNKVY 180
DGCC7710        ELTYGAGTMKENQVMGGENNYSLASHHIFGIAGASDMLFSPIDRAKNGMKIYLTDKNKVY 180
DGCC7796        ELTYGAGTMKENQVMGGENNYSLASHHIFGIAGASDMLFSPIDRAKNGMKIYLTDKNKVY 180
DGCC7854        ELTYGAGTMKENQVMGGENNYSLASHHIFGIAGASDMLFSPIDRAKNGMKIYLTDKNKVY 180
DGCC8014        ELTYGAGTMKENQVMGGENNYSLASHHIFGIAGASDMLFSPIDRAKNGMKIYLTDKNKVY 180
DGCC7909        ELTXGAGTMKENQVMGGENNYSLASHHIFGIAGASDMLFSPIDRAKNGMKIYLTDKNKVY 180
DGCC2058        ELTYGAGTMKENQVMGGENNYSLASHHIFGIAGASDMLFSPIDRAKNGMKIYLTDKNKVY 180
DGCC7785        XLTXGAGTMKENQVMGGENSYSLASHHIFGIAGASDMLFSPIDRAKNGMKIYLTDKNKVY 180
DGCC47          ELTXGAGTMKENQVMGGENSYSLASHHIFGIAGASDMLFSPIDRAKNGMKIYLTDKNKVY 180
DGCC7773        ELTXGAGTMKENQVMGGENSYSLASHHIFGIAGASDMLFSPIDRAKNGMKIYLTDKNKVY 180
CNRZ1066        ELTXGAGTMKENQVMGGENSYSLASHHIFGIAGASDMLFSPIDRAKNGMKIYLTDKNKVY 180
LMG18311        ELTXGAGTMKENQVMGGENNYSLASHHIFGIAGASDMLFSPIDRAKNGMKIYLTDKNKVY 180
DGCC7666        ELTXGAGTMKENQVMGGENNYSLASHHIFGIAGASDMLFSPIDRAKNGMKIYLTDKNKVY 180
                 ** ***************.*********************:****************:*

LMD-9           TYVISEVKIVQPTEVAVVDDTPGKSEVTLVTCTDAEATQRTIVKGNLESQVDFDKASSDI 240
DGCC7853        TYVISEVKIVQPTEVAVVDDTPGKSEVTLVTCTDAEATQRTIVKGNLESQVDFDKASSDI 240
DGCC7856        TYVISEVKIVQPTEVAVVDDTPGKSEVTLVTCTDAEATQRTIVKGNLESQVDFDKASSDI 240
DGCC855         TYVISEVKIVQPTEVAVVDDTPGKSEVTLVTCTDAEATQRTIVKGNLESQVDFDKASSDI 240
DGCC7879        TYVISEVKIVQPTEVAVVDDTPGKSEVTLVTCTDAEATQRTIVKGNLESQVDFDKASSDI 240
DGCC7984        TYVISEVKIVQPTEVAVVDDTPGKSEVTLVTCTDAEATQRTIVKGNLESQVDFDKASSDI 240
DGCC2057        TYVISEVKIVQPTEVAVVDDTPGKSEVTLVTCTDAEATQRTVVKGNLESQVDFDKASSDI 240
DGCC7766        TYVISEVKIVQPTEVAVVDDTPGKSEVTLVTCTDAEATQRTVVKGNLESQVDFDKASSNI 240
DGCC7790        TYVISEVKIVQPTEVALVDNTPGKSEVTLVTCTDAEATQRTVVKGNLESQIDFDKASSNI 240
DGCC9791        TYVISEVKIVQPTEVAVVDNTPGKSEVTLVTCTDAEATQRTVVKGNLESQIDFDKASSNI 240
DGCC7891        TYVISEVKIVQPTEVAVVDDTPGKSEVTLVTCTDAEATQRTVVKGNLESQVDFDKASSNI 240
ND03            TYVISEVKIVQPTEVAVVDDTPGKSEVTLVTCTDAEATQRTVVKGNLESQVDFDKASSNI 240
DGCC7809        TYVISEVKIVQPTEVAVVDDTPGKSEVTLVTCTDAEATQRTVVKGNLESQVDFDKASSDI 240
DGCC7694        TYVISEVKIVQPTEVAVVDDTPGKSEVTLVTCTDAEATQRTVVKGNLESQVDFDKASSDI 240
DGCC7710        TYVISEVKIVQPTEVAVVDDTPGKSEVTLVTCTDAEATQRTVVKGNLESQVDFDKASSDI 240
DGCC7796        TYVISEVKIVQPTEVAVVDDTPGKSEVTLVTCTDAEATQRTVVKGNLESQVDFDKASSDI 240
DGCC7854        TYVISEVKIVQPTEVAVVDDTPGKSEVTLVTCTDAEATQRTVVKGNLESQVDFDKASSDI 240
DGCC8014        TYVISEVKIVQPTEVAVVDDTPGKSEVTLVTCTDAEATQRTVVKGNLESQVDFDKASSDI 240
DGCC7909        TYVISEVKIVQPTEVAVVDDTPGKSEVTLVTCTDAEATQRTVVKGNLESQVDFDKASSDI 240
DGCC2058        TYVISEVKIVQPTEVAVVDDTPGKSEVTLVTCTDAEATQRTVVKGNLESQVDFDKASSDI 240
DGCC7785        TYVISEVKIVQPTEVAVVDDTPGKSEVTLVTCTDAEATQRTVVKGNLESQVDFDKASSNI 240
DGCC47          TYVISEVKIVQPTEVAVVDDTPGKSEVTLVTCTDAEATQRTVVKGNLESQVDFDKASSNI 240
DGCC7773        TYVISEVKIVQPTEVAVVDDTPGKSEVTLVTCTDAEATQRTVVKGNLESQVDFDKASSNI 240
CNRZ1066        TYVISEVKIVQPTEVAVVDDTPGKSEVTLVTCTDAEATQRTVVKGNLESQVDFDKASSNI 240
LMG18311        TYVISEVKIVQPTEVAVVDDTPGKSEVTLVTCTDAEATQRTVVKGNLESQVDFDKASSNI 240
DGCC7666        TYVISEVKIVQPTEVAVVDDTPGKSEVTLVTCTDAEATQRTVVKGNLESQVDFDKASSNI 240
                ****************:**:*********************:********:*******:*

LMD-9           LEAFNKSYNQFQS 253
DGCC7853        LEAFNKSYNQFQS 253
DGCC7856        LEAFNKSYNQFQS 253
DGCC855         LEAFNKSYNQFQS 253
DGCC7879        LEAFNKSYNQFQS 253
DGCC7984        LEAFNKSYNQFQS 253
DGCC2057        LEDFNKSYNQFQS 253
DGCC7766        LEDFNKSYNQFQS 253
DGCC7790        LEDFNKSYNQFQS 253
DGCC9791        LEDFNKSYNQFQS 253
DGCC7891        LEDFNKSYNQFQS 253
ND03            LEDFNKSYNQFQS 253
DGCC7809        LEDFNKSYNQFQS 253
DGCC7694        LEDFNKSYNQFQS 253
DGCC7710        LEDFNKSYNQFQS 253
DGCC7796        LEDFNKSYNQFQS 253
DGCC7854        LEDFNKSYNQFQS 253
DGCC8014        LEDFNKSYNQFQS 253
DGCC7909        LEDFNKSYNQFQS 253
DGCC2058        LEDFNKSYNQFQS 253
DGCC7785        LEDFNKSYNQFQS 253
DGCC47          LEDFNKSYNQFQS 253
DGCC7773        LEDFNKSYNQFQS 253
CNRZ1066        LEDFNKSYNQFQS 253
```

```
LMG18311        LEDFNKSYNQFQS 253
DGCC7666        LEDFNKSYNQFQS 253
                ** **********
```

Figure S4. Multiple sequence alignment of PrtS from *S. thermophilus*. Sequence alignment was performed with ClustalW2 package with default parameters (http://www.ebi.ac.uk/Tools/msa/clustalw2/). The Y123STOP mutation (X) is highlighted in red and the three catalytic residues (H147, C212 and R220) are highlighted in yellow. Genebank accession numbers: *S. thermophilus* LMD-9, YP_820636.1; ND03, ADQ63270.

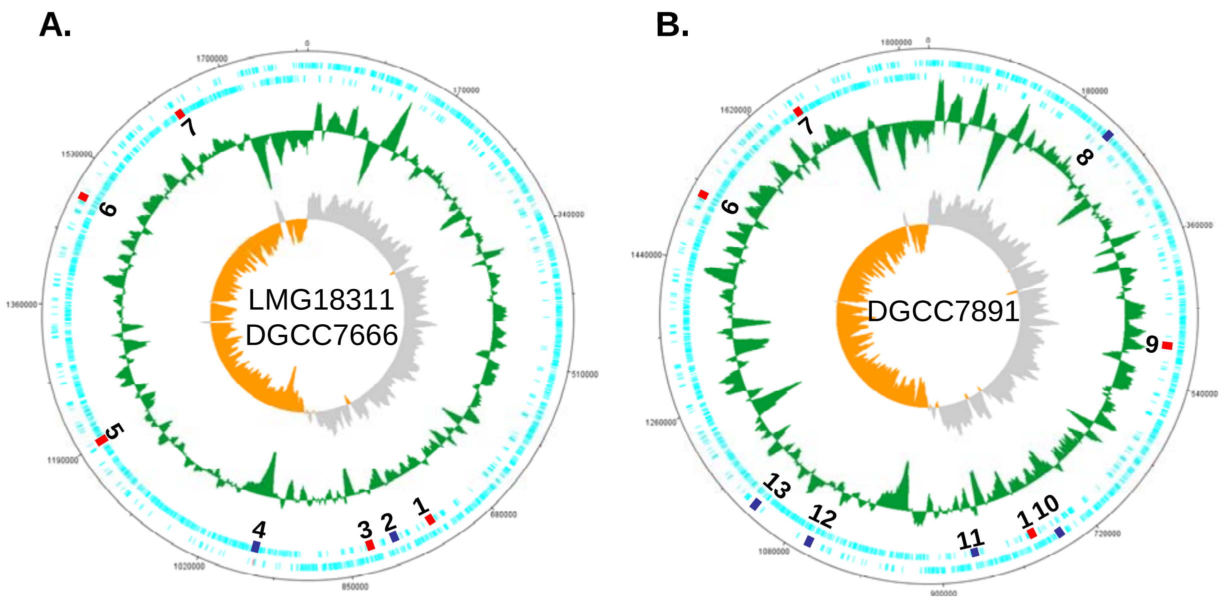

Figure S5. Schematic circular diagram of (A) LMG18311 and DGCC7666 and (B) DGCC7891 genomes, mapped with IS*Sth1* and IS*1167*. The insertion sites of the *prtS* island in PrtS-positive DGCC7666 strains are indicated on the LMG18311 diagram. Key for the circular diagram (outer to inner): scale (in Mb); annotated CDSs coloured in light blue are shown on a pair of concentric circles, representing both coding strands; red, IS*Sth1* ; blue, IS*1167* elements; the number correspond to IS*Sth1* and IS*1167* loci: 1 to *ciaH-rpsT*; 2 to *stu0861-stu0868*; 3 to *topA-stu0900*; 4 to *stu1089-stu1075*; 5 to *msrA1-brnQ*; 6 to *blpT-stu1680*; 7 to *galU-stu1836*; 8 to *STND0227-STND0229*; 9 to *STND0510-STND0513*; 10 to *STND0823-STND0825*; 11 to *STND0900-STND0902*; 12 to *STND1130-STND1132*; 13 to *STND1212-STND1214*; GC content of the chromosome, green; GC skew of the chromosome, grey and orange. Genome visualisation was performed with DNAPlotter (http://www.sanger.ac.uk/resources/software/dnaplotter/).

References

1. Bonifait L, Gottschalk M, Grenier D: **Cell surface characteristics of nontypeable isolates of Streptococcus suis.** *FEMS Microbiol Lett* 2010, **311:** 160-166.

2. Fernandez-Espla MD, Garault P, Monnet V, Rul F: ***Streptococcus thermophilus* cell wall-anchored proteinase: release, purification, and biochemical and genetic characterization.** *Appl Environ Microbiol* 2000, **66:** 4772-4778.
